# Molecular Insight into Drug Resistance Mechanism Conferred by Aberrant *PIK3CD* Splice Variant in African American Prostate Cancer

**DOI:** 10.3390/cancers15041337

**Published:** 2023-02-20

**Authors:** Siyoung Ha, Bi-Dar Wang

**Affiliations:** 1 Department of Pharmaceutical Sciences, University of Maryland Eastern Shore School of Pharmacy and Health Professions, Princess Anne, MD 21853, USA; 2 Hormone Related Cancers Program, University of Maryland Greenebaum Comprehensive Cancer Center, Baltimore, MD 21201, USA

**Keywords:** aberrant *PIK3CD* splice variant, prostate cancer disparities, Idelalisib resistance, molecular docking, SRSF2-mediated splicing, synergistic drug therapy

## Abstract

**Simple Summary:**

Higher incidence and mortality rates were observed in African American vs. European American prostate cancers. Alternative mRNA splicing has been suggested as an oncogenic driver in aggressive cancers as well as cancer disparities. Herein, we show that aberrant *PIK3CD-S* splice variant is overexpressed in aggressive African American prostate cancer, and its resulting PI3Kδ-S isoform is resistant to PI3Kδ inhibitors, such as Idelalisib and Seletalisib. Molecular modeling and functional validations implicate that a structural change due to exon 20 skipping greatly reduces the affinity between drugs and PI3Kδ-S isoform. Further targeting alternative splicing mechanism drastically sensitizes Idelalisib/Seletalisib-resistant prostate cancers to PI3Kδ inhibitors. These results have highlighted the therapeutic potential of modulating RNA splicing mechanism to overcome drug resistance in aggressive cancers.

**Abstract:**

Targeting PI3Kδ has emerged as a promising therapy for hematologic and non-hematologic malignancies. Previously, we identified an oncogenic splice variant, *PIK3CD-S*, conferring Idelalisib resistance in African American (AA) prostate cancer (PCa). In the current study, we employed a comprehensive analysis combining molecular biology, biochemistry, histology, in silico simulation, and in vitro functional assays to investigate the *PIK3CD-S* expression profiles in PCa samples and to elucidate the drug resistance mechanism mediated by PI3Kδ-S (encoded by *PIK3CD-S*). The immunohistochemistry, RT-PCR, and Western blot assays first confirmed that PI3Kδ-S is highly expressed in AA PCa. Compared with PCa expressing the full-length PI3Kδ-L, PCa expressing PI3Kδ-S exhibits enhanced drug resistance properties, including a higher cell viability, more antiapoptotic and invasive capacities, and constitutively activated PI3K/AKT signaling, in the presence of PI3Kδ/PI3K inhibitors (Idelalisib, Seletalisib, Wortmannin, and Dactolisib). Molecular docking, ATP-competitive assays, and PI3 kinase assays have further indicated a drastically reduced affinity of PI3Kδ inhibitors with PI3Kδ-S vs. PI3Kδ-L, attributed to the lack of core binding residues in the PI3Kδ-S catalytic domain. Additionally, SRSF2 has been identified as a critical splicing factor mediating exon 20 skipping in *PIK3CD* pre-mRNA. The inhibition of the SRSF2 activity by SRPIN340 successfully sensitizes AA PCa cells to PI3Kδ inhibitors, suggesting a novel therapeutic option for Idelalisib-resistant tumors.

## 1. Introduction

Prostate cancer (PCa) is the most frequently diagnosed and second leading cause of cancer death among American men. Particularly, African American (AA) men demonstrate a 1.7-fold higher incidence rate and a 2.3-fold higher mortality rate compared to the European American (EA) counterparts [1]. Emerging evidence has shown that, besides the socioeconomic factors, intrinsic genetic factors account for part of the observed disparities. Differential epigenomes (DNA methylation patterns [2,3,4] and microRNA regulatory networks [5,6,7,8]), transcriptomes [9,10,11,12,13], and mRNA splicing landscapes [14] have been revealed as critical genetic factors that differentiate the oncogenic properties of AA PCa from EA PCa.

Deep sequencing studies have suggested that more than 95% of human genes have undergone alternative splicing at the pre-mRNA level [15,16], oftentimes resulting in protein isoforms with distinct, or even completely opposite functions. Intriguingly, tumor cells demonstrate more active alternative splicing programs (with up to 30% more mRNA splicing events) than the normal cells [17]. Accumulating studies have implicated that the alternative splicing of oncogenes and tumor suppressor genes may play critical functional roles in the development and progression of cancers, including PCa [18,19,20]. Previously, we have identified >2500 differential splicing (DS) events between AA PCa and EA PCa. Among the identified DS events/genes, 71.8% are functionally related to cancer diseases. Several oncogenes/tumor suppressor genes, such as *PIK3CD, FGFR3, TSC2, ITGA4, MET, NF1, BAK1,* and *RASGRP2*, were identified and verified with DS events occurring between AA and EA PCa [14]. Notably, the overexpression of aberrant splice variants of *PIK3CD-S* (where exon 20 is skipped) and *FGFR3-S* (where exon 14 is skipped) have been implicated as critical oncogenic elements to promote AA PCa aggressiveness and drug resistance [14,21].

PI3Kδ is frequently upregulated in B-cell malignancies, such as in chronic lymphocytic leukemia (CLL), multiple myeloma (MM), lymphoma, non-Hodgkin’s lymphoma (NHL), acute myeloid leukemia (AML), and acute promyelocytic leukemia (APL) [22]. Accumulating studies have further indicated that PI3Kδ is also highly expressed in solid tumors, including colon, lung and breast cancers, melanoma, neuroblastoma, and PCa [23,24]. Targeting PI3Kδ, therefore, has emerged as a promising therapeutic option in cancers of hematologic and non-hematologic origins. Idelalisib is a first-in-class, orally bioavailable small-molecule inhibitor approved for treating relapsed/refractory CLL, follicular lymphoma, and small lymphocytic lymphoma [25,26]. Mechanistically, Idelalisib is an ATP-competitive inhibitor specifically targeting PI3Kδ, with a potent efficacy for PI3Kδ-expressing cancers. Despite the favorable efficacy of this agent against B-cell malignancies, resistance to Idelalisib remains a clinical challenge/concern for CLL therapy. However, the molecular mechanism underlying the Idelalisib resistance remains unclear. In the present study, we hypothesized that the aberrant mRNA splicing of *PIK3CD* (that encodes PI3Kδ splice isoform) is one of the driving mechanisms conferring resistance to Idelalisib therapy. A panel of comprehensive analyses combining immunohistochemistry, biochemistry, molecular biology, in silico molecular modeling, and in vitro functional assays has been applied to validate our hypothesis. Specifically, IHC assays and a Western blot and RT-PCR analysis were performed to examine the expression levels of PI3Kδ-L and PI3K-S isoforms (at the protein and RNA levels) in PCa specimens and cell lines derived from AA and EA patients. Secondly, four small-molecule inhibitors (Idelalisib, Seletalisib, Wortmannin, and Dactolisib) were used to test the drug efficacies against the PCa cells expressing *PIK3CD-L* (encoding full-length PI3Kδ) and the *PIK3CD-S* splice variant. Similar to Idelalisib, Seletalisib is a quinolone-based, orally available small-molecule inhibitor with selective inhibitory activity against PI3Kδ [27]. Wortmannin is one of the earliest generations of the ATP-competitive PI3K inhibitor, with broad inhibitory efficacies against all PI3Kα, PI3Kβ, PI3Kδ, and PI3Kγ isoforms [28]. Dactolisib is an imidazoquinoline derivative exhibiting dual inhibitory activities against PI3Kδ and mTOR, and the combination therapy with Everolimus is under clinical trial for the treatment of solid tumors [29]. Thirdly, a series of in vitro functional assays (including MTT, invasion, apoptosis, ATP/drug-competitive assays, and cell-free kinase assays) were conducted to assess whether the PI3Kδ-S splice isoform is more resistant to these pan-PI3Kδ and PI3Kδ-specific inhibitors than the full-length PI3Kδ-L. Finally, critical splicing regulators (such as SRSF2 and HNRNPF, upregulated in AA PCa vs. EA PCa) were evaluated for their involvement in the *PIK3CD-S* synthesis and functional link with the PI3Kδ-S-mediated drug resistance in AA PCa. To date, this is the first attempt to decipher the molecular mechanism underlying the resistance to PI3Kδ and pan-PI3K inhibitors in PCa. This study aims to provide molecular insights into the interactions/competitions of the inhibitors and ATP with PI3Kδ-L and PI3Kδ-S, facilitating our understanding of the molecular mechanisms underlying Idelalisib resistance and further developing a novel therapeutic strategy for treating resistant AA PCa or PI3Kδ-S overexpressing tumors with hematologic and non-hematologic origins.

## 2. Methods

### 2.1. Cell Culture

PCa cell lines 22Rv1, LNCaP, PC-3, RC77 T/E, and MDA PCa 2b were used in this study. 22Rv1 represents a castration-resistant EA PCa cell line, while LNCaP and PC-3 were derived from EA PCa patients with lymph node and bone metastases, respectively. RC77 T/E is a primary PCa cell line derived from AA patient (kindly provided by Dr. Johng Rhim at CPDR, Rockville, MD, USA), while MDA PCa 2b is a cell line derived from bone metastasis of an AA PCa patient. Except RC77 T/E, all the PCa cell lines were purchased from American Type Culture Collection (ATCC, Manassas, VA, USA). The EA PCa cell lines LNCaP and 22Rv1 were cultured in RPMI with 10% fetal bovine serum (FBS), while PC-3 was cultured in DMEM with 10% FBS. AA PCa cell lines RC77 T/E and MDA PCa 2b were grown in Keratinocyte SFM with human recombinant EGF1-53 and BPE, and BRFF-HPC1 with 20% FBS, respectively. All the cell lines were maintained at 37 °C in a 5% CO_2_ incubator.

### 2.2. RT-PCR Assay

Total RNA samples were purified from the PCa cells using miRNeasy mini kit (Qiagen, Germantown, MD, USA) according to the manufacturer’s protocol. Purified RNA samples were reversely transcribed to cDNA using iScript reverse transcription supermix (Bio-Rad, Hercules, CA, USA). Briefly, 1 μg of RNA was mixed with 4 μL of iScript RT supermix and RNase-free water to total volume of 20 µL. The reverse transcription reactions were performed at follows: 25 °C for 5 min, 46 °C for 50 min, then 95 °C for 1 min. The resulting cDNA samples were used for PCR reactions to examine *PIK3CD-L* and *PIK3CD-S* expression levels, and *EIF1AX* was used as an endogenous control. The primers for PCR reactions are listed on Supplementary Table S1.

### 2.3. Immunohistochemistry (IHC) Assay

Tissue microarrays (TMAs) containing prostate tissues from 3 healthy AAs and 3 healthy EAs, PCa samples, and adjacent normal tissues derived from 40–50 AA PCa and EA PC patients were used for the IHC staining of PI3Kδ-S and SRSF2 expression levels. IHC assays were performed using the standard protocol established in our lab [6,30]. Briefly, TMA slides were deparaffinized in xylene and xylene/alcohol (1:1) solution, then followed by serial rehydration using graded alcohols (100%, 95%, 70%, and 50% of alcohol, respectively) to distilled water. EnVision FLEX target retrieval solution from Agilent technologies (Carpinteria, CA, USA) was used according to the manufacturer’s protocol. Specifically, antigen retrieval was performed in microwave (full power for 5 min, and 20% power for an additional 20 min), followed by cooling down for 30 min at room temperature. Peroxidase block was added dropwise and incubated for 30 min, and the slides were then washed with 1 × PBS twice for 5 min followed by adding blocking buffer (2.5% BSA in 1 × PBS) and incubating for 30 min at room temperature. TMAs were then incubated with the primary antibody (1:200 dilutions in 2.5% BSA/1 × PBS) at 4 °C overnight. After washing with 1 × PBS twice for 5 min, the TMAs were incubated with HRP-conjugated secondary antibody (Dako, Carpinteria, CA, USA) for 1h, and HRP was detected by DAB-chromogen (Dako, Carpinteria, CA, USA), then counterstained with Mayer’s hematoxylin (Sigma, St. Louis, MO, USA). IHC images were captured using Pannormic Midi Digital Scanner (3DHISTECH Ltd., Budapest, Hungary) and viewed using CaseViewer software (3DHISTECH, Budapest, Hungary). The analysis and quantification of PI3Kδ and SRSF2 intensities were performed using ImageJ software (NIH, Bethesda, Rockville, MD, USA) as described in our previous study [30]. The statistical analysis was performed using ANOVA with post hoc Tukey’s test for multiple comparisons. The PI3Kδ-S antibody was a custom-made rabbit polyclonal antibody from Thermo Fisher Scientific (Waltham, MA, USA), and SRSF2 antibody was a rabbit polyclonal antibody purchased from Abcam (Cambridge, UK).

### 2.4. MTT (Cell Viability) Assay

For the measurement of cell viability, 22Rv1, LNCaP, PC-3, RC77 T/E, and MDA PCa 2b cells were seeded at density 5 × 10^3^ cells/well in 96-well plates and grown for 24 h. The EA PCa LNCaP cells transfected with *PIK3CD-L* or *PIK3CD-S,* and AA PCa MDA PCa 2b stably expressing *PIK3CD-L* or *PIK3CD-S* were also seeded at density 5 × 10^3^ cells/well in 96-well plates. Then, the cell cultures were replaced with fresh media, and the PCa cells were treated with four small-molecule inhibitors (Idelalisib, Seletalisib, Wortmannin, and Dactolisib) at concentrations ranging from 0 to 100 μM. MTT assays were then conducted to examine cell viabilities of the tested PCa cells after drug treatment for 48 h. Specifically, MTT assay reagent was added to the cells at a final concentration of 0.5 mg/mL in phenol-free media and incubated for 3 h in a 5% CO_2_ incubator at 37 °C. Then, the samples were detected by the Mutiskan FC microplate photometer (Thermos Fisher Scientific, Waltham, MA, USA) at the wavelength of 570 nm, then the data were analyzed using the GraphPad Prism 9 program (GraphPad Software, La Jolla, CA, USA) for graphing and statistical analysis.

### 2.5. Cell Apoptosis Assay

LNCaP and MDA PCa 2b cells overexpressing *PIK3CD-L* or *PIK3CD-S* were subjected to apoptosis assays for evaluating the apoptosis capacities upon different drug treatments. The PCa cells were seeded in 96-well plates at initial cell density of (5 × 10^3^ cells/well) and were grown for 24 h, then were treated with four small-molecule inhibitors (Idelalisib, Seletalisib, Wortmannin, and Dactolisib) at concentrations of 0, 1, 10, and 25 μM. After drug treatment for 48 h, the PCa cells were harvested for the apoptosis assays using Apo-ONE Caspase-3/7 Assay Kit (Promega Corporation, Madison, WI, USA) according to the protocol described by the manufacturer. The apoptosis activities were measured by detecting fluorescence signals at wavelengths of 498 nm and 521 nm (for excitation and emission, respectively) using Biotek Synergy HT Microplate Reader (BioTek, Winooski, VT, USA).

### 2.6. Invasion Assay

The effects of Idelalisib, Seletalisib, Wortmannin, and Dactolisib at different concentrations (0, 1, and 25 μM) on inhibiting invasion abilities of *PIK3CD-L*- and *PIK3CD-S*-expressing LNCaP and MDA PCa 2b cells were evaluated using Corning Matrigel Invasion Chamber 8.0 Micron (Corning, Cambridge, MA, USA), according to manufacturer’s protocol. Specifically, 2 × 10^4^ cells in serum-reduced medium were seeded in the upper chamber with four small-molecule inhibitors and were cultured overnight in a 5% CO_2_ at 37 °C. After growing for 24 h, the PCa cells were subjected to invasion assays. The non-invading cells were wiped off from the top membrane of the invasion chamber using cotton swabs, and the cells invading through the bottom membrane were fixed and stained with Wright-Giemsa quick stain (Cambridge Diagnostic Products, Fort Lauderdale, FL, USA), washed with water, and allowed to dry for counting the number of invading cells to determine the invasive capacities of PCa cells in different drug treatment groups.

### 2.7. Western Blot Analysis

Wild-type 22Rv1, LNCaP, PC-3, RC77 T/E, and MDA PCa 2b without drug treatments were grown and harvested for Western blot analysis. Moreover, MDA PCa 2b cells stably overexpressing *PIK3CD-L* and *PIK3CD-S* were grown and treated with four small-molecule inhibitors (at 0, 1, 10, and 25 μM) for 48 h, then the cells were harvested and subjected to Western blot analysis. The Western blot assays were performed using standardized protocol in our lab as previously described [5,14,30]. Briefly, the cells were collected, and total proteins were extracted using M-PER Mammalian Protein Extraction Reagent (Thermo Fisher Scientific, Waltham, MA, USA) with protease and phosphatase inhibitor cocktail (Thermo Fisher Scientific, Waltham, MA, USA). Quantification of protein concentrations from individual protein samples were determined using BCA assay kit (Thermo Fisher Scientific, Waltham, MA, USA). Bolt 4–12% and 8% Bis-Tris mini protein gels (Thermo Fisher Scientific, Waltham, MA, USA) were used for running protein gel electrophoresis. The primary antibodies used in the study were rabbit monoclonal antibodies against pAKT, AKT, pmTOR, mTOR, pS6, S6, and β-actin from Cell Signaling Technology (Danvers, MA, USA), polyclonal rabbit antibody against PI3Kδ-S from Thermo Fisher Scientific (Waltham, MA, USA), and monoclonal mouse antibody against PI3Kδ from Santa Cruz Biotechnology (Dallas, TX, USA). The secondary antibody used was anti-rabbit IgG-HRP and anti-mouse IgG-HRP antibodies purchased from Thermo Fisher Scientific (Waltham, MA, USA).

### 2.8. Molecular Docking

Molecular modeling for interactions of the small-molecule inhibitors with PI3Kδ-L or PI3Kδ-S splice isoform were performed using the AutoDock vina (https://vina.scripps.edu/, accessed since 1 September 2021) and the predicted protein/drug complexes were visualized with the PyMOL software (https://pymol.org/2/, accessed since 1 September 2021). The three-dimensional (3D) structures of Idelalisib, Seletalisib, Wortmannin, and Dactolisib were drawn by ChemDraw 20.1.1, and then the structures were optimized and used as input structures for docking modeling. The amino acid sequences reflecting the kinase domain of full-length PI3Kδ (PI3Kδ-L) and PI3Kδ-S splice isoform are listed in Supplementary Table S2. These amino acid sequences were entered into SWISS-MODEL (http://www.expasy.org/swissmod/SWISS-MODEL.html, accessed since 1 September 2021) for homology crystal structure models. A binding pocket was selected by the active site residues involved in the binding of the ATP with PI3Kδ and a grid-based method was used for energy calculation of the flexible ligand with the target protein. RMSD measurements were calculated by finding the square root of the mean square error using Autodock vina and the top energy models were analyzed/visualized by PyMOL.

### 2.9. ATP-Competitive Binding Assay

For understand the competition of small-molecule inhibitors with ATP to specifically bind to PI3Kδ-L or PI3Kδ-S splice isoform, we first prepared purified recombinant protein of PI3Kδ-L and PI3Kδ-S for the ATP-competitive binding assays. To validate the molecular docking results, drug/ATP-competitive assays were conducted. Specifically, purified PI3Kδ-L or PI3Kδ-S isoform was incubated with small-molecule inhibitor and ATP, and the ATP intensity in the final eluted sample (containing PI3Kδ-L/drug and PI3Kδ-L/ATP, or PI3Kδ-S/drug and PI3Kδ-S/ATP) was measured using ATP Colorimetric/Fluorometric Assay Kit (Sigma-Aldrich, Louis, MO, USA). pcDNA3.1-*PIK3CD-L*-V5-HIS and pcDNA3.1-PIK3CD-S-V5/HIS were the plasmids containing the coding sequences of *PIK3CD-L* and *PIK3CD-S* with V5 and 6 × His tags. These two plasmids were transfected into HEK-293 cell with Lipofectamine 3000 Reagent (Life Technologies, Carlsbad, CA, USA) and incubated in a 5% CO_2_, at 37 °C. After 16 h, cells were harvested and total proteins were extracted using M-PER reagent with protease and phosphatase inhibitor cocktail, and the His-tagged PI3Kδ-L and PI3Kδ-S isoforms were purified using 1-mL His Gravi Trap column (GE Healthcare, Chicago, IL, USA). The purified proteins were then further dialyzed with 10 mM Tris/HCL (pH7.4) at 4 °C, and stored at −80 °C. The purified proteins were confirmed by Coomassie blue staining and Western blots with anti-Histidine and anti-PI3Kδ antibodies from Cell Signaling Technology (Danvers, MA, USA). For each ATP assay, 10 mM of purified His-tagged PI3Kδ-L or PI3Kδ-S was first bounded to the His Trap column, then the small-molecule inhibitor (10 mM) and ATP (1 mM) were incubated with the nickel column-bound PI3Kδ-L or PI3Kδ-S. The reaction mixtures were spun down, washed, and eluted using 250 mM of imidazole. Finally, the fluorescence signals of the eluted samples were detected at the wavelengths at 535 nm (for excitation) and 587 nm (for emission) using BioTek Synergy HT microplate reader (BioTek Instruments, Winooski, VT, USA).

### 2.10. Cell-Free Kinase Activity Assay

The PCa cell lines (LNCaP, PC-3, and MDA PCa 2b) transfected with pcDNA3.1-*PIK3CD-L*-V5-HIS or pcDNA3.1-*PIK3CD-S*-V5-HIS plasmid were harvested for purification of PI3Kδ-L and PI3Kδ-S isoforms. The His-tagged PI3Kδ-L and PI3Kδ-S were purified using HisPur Ni-NTA spin columns (Thermo Fisher Scientific, Waltham, MA, USA). The Ni-NTA column-bound PI3Kδ-L-6 × His and PI3Kδ-S-6 × His were washed with 1 × PBS twice, then were eluted using the elution buffer (20 mM Tris/HCl, 200 mM sodium chloride, 250 mM imidazole, pH7.5). The flow-through, wash, and elute samples were collected for Western blot analysis to evaluate the purification efficiencies (Supplementary Figure S3). The His-tagged PI3Kδ-L and PI3Kδ-S isoforms were incubated with four small-molecule inhibitors (Idelelisib, Seletalisib, Wortmannin, and Dactolisib) for 30 min at room temperature, and the protein/drug mixtures were then subjected to kinase activity assays using PI3 kinase activity/inhibitor assay kit (Millipore, Burlington, MA, USA) according to the protocol provided by the manufacturer.

### 2.11. RNA Pulldown Assay

The negative control RNA and RNA containing SFSR2 and HRNPF putative motifs in upstream flanking intron (intron 19) of *PIK3CD* pre-mRNA was labeled with biotin using Pierce’s RNA 3′ end biotinylation kit (Waltham, MA, USA) according to the manufacturer’s protocol. A total of 50 μL of streptavidin magnet beads was incubated with 50 pmol of biotin-labeled RNA sample in a total volume of 100 μL of 1 × RNA capture buffer. After incubation for 30 min at room temperature with agitation, the biotin-labeled RNA/streptavidin magnetic beads were washed twice with 20 mM Tris (pH7.5) and resuspended in 100 μL of 1 × protein-RNA-binding buffer. A total of 100 μL of PCa lysate-containing RNA-binding buffer was added and incubated overnight at 4 °C with rotation. The RNA-binding protein complexes were then washed twice with 1 × wash buffer and eluted with 50 μL of elution buffer. The eluted RNA-protein complex samples were then subjected to Western blot analysis using anti-SRSF2 (Abcam, Cambridge, UK) or anti-HNRNPF antibody (Santa Cruz Biotechnology, Dallas, TX, USA).

### 2.12. RNA Immunoprecipitation (RIP) Assay

Synthesized RNA fragment containing putative SRSF2 and binding motifs of intron 19 was used as RNA template for RIP and RNA pulldown assays. Nuclear protein extract was prepared using the subcellular protein fractionation kit (Thermo Fisher Scientific, Waltham, MA, USA). The RIP assays were performed using Magna RIP RNA-binding protein precipitation kit according to the protocol described by the manufacturer (MilliporeSigma, Burlington, MA, USA). Briefly, nuclear protein extracts from MDA PCa 2b were incubated with the synthesized RNA fragment containing putative SRSF2-binding motifs. After incubation at room temperature for 30 min, 5 µg of IgG or SRSF2 antibody was added to the RNA/nuclear protein mixture and incubated with rotation at room temperature for 1 h. A total of 50 µL of magnet beads was added to each of the RNA/nuclear protein/antibody mixture for incubation at 4 °C overnight. The immunoprecipitated protein/RNA complexes were then washed with RIP wash buffer five times, then resuspended in 100 μL of RIP buffer. The suspension was subjected to proteinase K/10%SDS digestion in total volume of 150 μL, and RNA was then purified for RT-PCR assays. The primer sequences used for the PCR reactions were listed in Supplementary Table S3.

## 3. Results

### 3.1. PIK3CD-S Splice Variant Is Overexpressed in AA PCa

**Figure 1:**
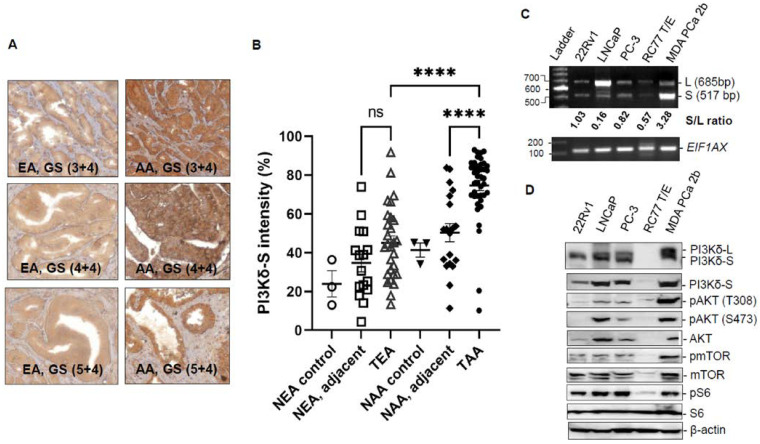
PI3Kδ-S splice isoform is overexpressed in AA PCa with highly activated AKT/mTOR signaling. (**A**) IHC staining of representative AA and EA PCa tissue samples with different Gleason Scores (GS). (**B**) Quantification of PI3Kδ-S intensities, based on IHC staining signals, of normal EA (NEA), normal adjacent and tumor samples from EA PCa (NEA, adjacent and TEA), normal AAs (NAA), normal adjacent and tumor samples from AA PCa (NAA, adjacent and TAA). **** *p*-value < 0.0001, based on one-way ANOVA with Tukey’s post hoc test. ns: not significant. (**C**) RT-PCR analysis of *PIK3CD-L* and *PIK3CD-S* expression levels in a panel of cell lines derived from EA PCa (22Rv1, LNCaP, and PC-3) and AA PCa (RC77 T/E and MDA PCa 2b). *EIF1AX* was used as an endogenous control. (**D**) Western blot analysis of PI3Kδ isoforms (L and S), pAKT, AKT, pmTOR, mTOR, pS6, S6, and β-actin in EA PCa (22Rv1, LNCaP, and PC-3) and AA PCa (RC77 T/E and MDA PCa 2b) cells.

Our previous genomic study has revealed that aberrant splice variants of *PIK3CD* were differentially expressed between AA PCa and EA PCa patient specimens [14]. To further validate the expression profiles and oncogenic properties of *PIK3CD-L* (encoding full-length PI3Kδ) and *PIK3CD-S* (splice variant missing exon 20, encoding a short splice isoform PI3Kδ-S), a TMA containing AA and EA PCa specimens and a panel of AA and EA PCa cell lines were subjected to IHC and Western blot assays. Specifically, an antibody specifically designed for detecting the PI3Kδ-S isoform was used in the IHC staining procedure. The TMA used for the IHC contains tumor and adjacent normal tissues derived from 40–50 EA and AA PCa patients and normal tissues derived from 3 EA and 3 AA healthy individuals. The IHC staining results have shown that AA PCa expressed a significantly higher PI3Kδ-S level than EA PCa. Moreover, the PI3Kδ-S is upregulated in AA PCa vs. AA adjacent normal (Figure 1A,B). These results confirmed that the PI3Kδ-S isoform is highly expressed in AA PCa vs. EA PCa, consistent with the exon array data and the RT-PCR data in our previous study [14]. Additionally, a panel of EA PCa (22Rv1, LNCaP and PC-3) and AA PCa (RC77 T/E and MDA PCa 2b) cell lines were used as in vitro cell line models for evaluating the expression profiles and oncogenic properties of *PIK3CD-L* and *PIK3CD-S* in EA and AA PCa at the RNA level. First, the RNA samples were purified from the EA and AA PCa cell lines and the purified RNA samples were subjected to RT-PCR validation. The RT-PCR results have demonstrated that the AA PCa cell line MDA PCa 2b (androgen-independent, bone metastasis PCa) expressed the highest level of the *PIK3CD-S* splice transcript compared to all other PCa cell lines. In contrast, the EA PCa cell line LNCaP (an androgen-dependent PCa derived from lymph node metastasis) predominately expressed the *PIK3CD-L* transcript. The other two androgen-independent EA PCa cell lines, 22Rv1 and PC-3, expressed both *PIK3CD-L* and *PIK3CD-S.* The AA PCa cell line RC77 T/E (derived from a primary tumor) expressed very low levels of the *PIK3CD-L* and *PIK3CD-S* transcripts. The *PIK3CD-S/PIK3CD-L* ratios (S/L ratios) of the 22Rv1, LNCaP, PC-3, RC77 T/E, and MDA PCa 2b were 1.03, 0.16, 0.82, 0.57, and 3.28, respectively (Figure 1C). Second, the protein lysates isolated from the EA and AA PCa cell lines were subjected to Western blot assays to evaluate the correlation between the *PIK3CD-S/PIK3CD-L* profiles and the regulation of the AKT/mTOR signaling. The PI3Kδ-L and PI3Kδ-S expression levels were consistent with the RT-PCR results shown in Figure 1C, where the AA PCa cell line MDA PCa 2b exhibited the highest level of PI3Kδ-S compared to the other PCa cell lines. Notably, the MDA PCa 2b cells also demonstrated the highest phosphorylation states of the AKT, mTOR, and S6 (pAKT-T308, pAKT-S473, pmTOR, and pS6), suggesting that the AKT/mTOR signaling is significantly upregulated in metastatic AA PCa (MDA PCa 2b, with highest *PIK3CD-S/PIK3CD-L* ratio) vs. metastatic EA PCa (22Rv1, LNCaP, and PC-3) and AA primary PCa RC77 T/E (Figure 1D). Taken together, our IHC, RT-PCR, and Western blot results have confirmed that the aberrant *PIK3CD-S* splice variant is upregulated in AA PCa vs. EA PCa at the mRNA and protein levels, and the higher PI3Kδ-S expression level enhanced the activation of the AKT/mTOR signaling in AA PCa.

### 3.2. AA PCa Cells Are More Resistant to PI3Kδ-Specific, Pan-PI3K, and PI3K/Mtor Dual Inhibitors

Our previous study suggested that the PI3Kδ-S isoform may have more oncogenic properties than the full-length PI3Kδ-L [14]. To assess whether the splice isoform PI3Kδ-S confers an enhanced drug resistance than PI3Kδ-L, MTT assays were performed to examine the drug inhibitory effects on a panel of PCa cell lines (22Rv1, LNCaP, PC-3, RC77 T/E, and MDa PCa 2b) expressing different *PIK3CD-S/PIK3CD-L* profiles. Specifically, the PCa cells were treated with four small-molecule inhibitors, including Idelalisib, Seletalisib, Wortmannin, and Dactolisib. Seletalisib and Idelalisib are ATP-competitive kinase inhibitors that target the phosphoinositide 3-kinase δ isoform (PI3Kδ) with high selectivity and potency, Wortmannin is a pan-PI3K inhibitor that targets PI3Kα, β, δ, and γ, while Dactolisib is a dual inhibitor simultaneously targeting PI3K and mTOR [25,27,28,29].

**Figure 2:**
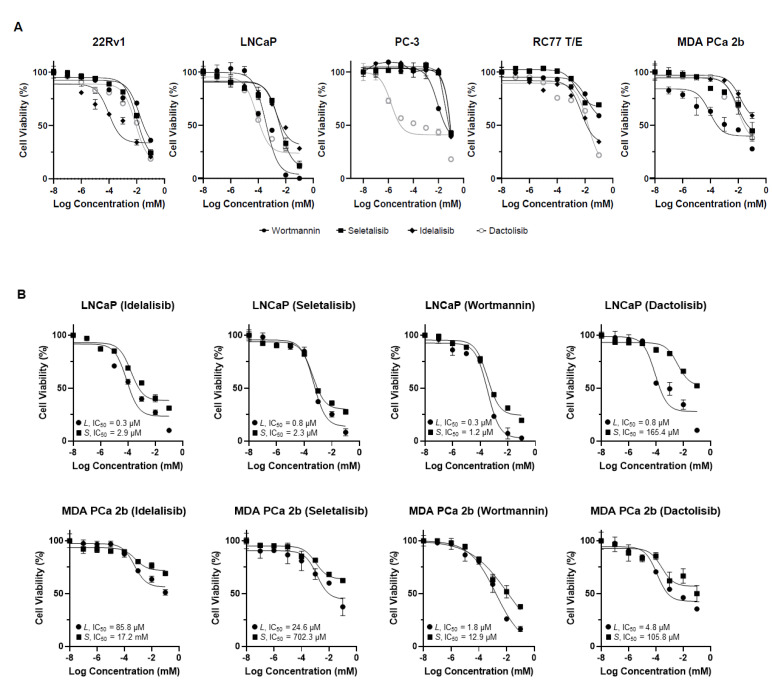
Cell viability curves of the PCa cells treated with PI3Kδ-specific, pan-PI3K, and PI3K/mTOR inhibitors by MTT assays. (**A**) Cell viability curves from EA PCa cell lines (22Rv1, LNCaP, and PC-3) and AA PCa cell lines (RC77 T/E and MDA PCa 2b) in presence of Idelalisib, Seletalisib, Wortmannin, and Dactolisib at concentrations from 0 to100 µM. (**B**) Cell viability curves from *PIK3CD-L*- and *PIK3CD-S*-expressing LNCaP and MDA PCa 2b cells in the presence of four drugs at concentrations from 0 to100 µM. All the MTT assay results (in A and B) were calculated based on six experimental repeats for each drug dose, and the data values represent mean ± SD. The IC_50_ values were determined from the viability curves by using GraphPad Prism 9 program.

We first observed that the drugs Idelalisib, Seletalisib, Wortmannin, and Dactolisib potently impaired the viability of the 22Rv1, LNCaP, and PC-3 (EA PCa) cells lines and the RC77 T/E and MDA PCa 2b (AA PCa) cell lines in a dose-dependent manner (Figure 2A). The IC_50_ values of Idelalisib, Seletalisib, Wortmannin, and Dactolisib were 1.1 μM, 15.7 μM, 36.2 μM, and 9.2 μM for 22Rv1; 8.2 μM, 3.7 μM, 0.4 μM, and 0.2 μM for LNCaP; 80.2 μM, 85.5 μM, 46.5 μM, and 0.2 μM for PC-3; 17.2 μM 176.7 μM, 121.3 μM, and 16.4 μM for RC77 T/E; and 123.2 μM, 53.1 μM, 1.4 μM, and 35.9 μM for MDA PCa 2b, respectively (Supplementary Table S4). Overall, the results showed that the AA PCa cell lines (RC77 T/E and MDA PCa 2b) appear to be more resistant to the drugs (generally with higher IC_50_ values, especially highly resistant to PI3Kδ-specific inhibitors Idelalisib and Seletalisib) than the EA PCa cell lines.

### 3.3. PCa Cells Expressing PIK3CD-S Splice Variant Exhibit Drug Resistance Phenotype When Treated with PI3Kδ-Specific, Pan-PI3K, and PI3K/Mtor Dual Inhibitors

Based on the MTT results from Figure 2, we hypothesized that *PIK3CD-S*, which is highly expressed in MDA PCa 2b, may represent a more oncogenic/drug-resistant splice variant compared to the *PIK3CD-L*. To further verify this hypothesis, the LNCaP and MDA PCa 2b cells expressing the differential *PIK3CD-L* or *PIK3CD-S* were treated with pan-PI3K and PI3Kδ-specific drugs to examine the drug efficacies against these PCa cells with distinct *PIK3CD-L* and *PIK3CD-S* expression profiles. Specifically, the LNCaP cells were first transfected with pcDNA3.1-*PIK3CD-L*-V5-HIS or pcDNA3.1-*PIK3CD-S*-V5-HIS plasmid for 16 h, and then the cells were treated with four small-molecule inhibitors (Idelalisib, Seletalisib, Wortmannin, and Dactolisib, at concentrations of 0–100 μM) for an additional 48 h. The MDA PCa 2b cells stably expressing *PIK3CD-L* or *PIK3CD-S* (Supplementary Figure S1) were grown and treated with the same small-molecule inhibitors (at concentrations of 0 to 100 μM). After drug treatments for 48 h, the *PIK3CD-L*- and *PIK3CD-S*-expressing MDA PCa 2b cells were subjected to MTT assays. Under the treatments of Idelalisib, Seletalisib, Wortmannin, and Dactolisib, the cell viabilities were significantly lower in the *PIK3CD-L*-expressing cells compared to the *PIK3CD-S*-expressing cells, in either the LNCaP or MDA PCa 2b background (Figure 2B). The IC_50_ values were 0.3 μM and 2.9 μM for Idelalisib, 0.8 μM and 2.3 μM for Seletalisib, 0.3 μM and 1.2 μM for Wortmannin, and 0.8 μM and 165.4 μM for Dactolisib in the *PIK3CD-L*- and *PIK3CD-S*-expressing LNCaP, respectively (Figure 2B, top panel). The IC_50_ values were 85.8 μM and 17.2 mM for Idelalisib, 24.6 μM and 702.3 μM for Seletalisib, 1.8 μM and 12.9 μM for Wortmannin, and 4.8 μM and 105.8 μM for Dactolisib in *PIK3CD-L*- and *PIK3CD-S*-expressing MDA PCa 2b, respectively (Figure 2B, bottom panel). Taken together, these MTT results strongly suggest that *PIK3CD-S*-overexpressing cells were more resistant to all the four drugs (with 4- to 207-fold higher IC_50_ values in the *PIK3CD-S*- vs. *PIK3CD-L*-expressing cells).

### 3.4. Overexpression of PIK3CD-S Splice Variant Enhances Antiapoptotic and Invasive Capacities in PCa Cells

**Figure 3:**
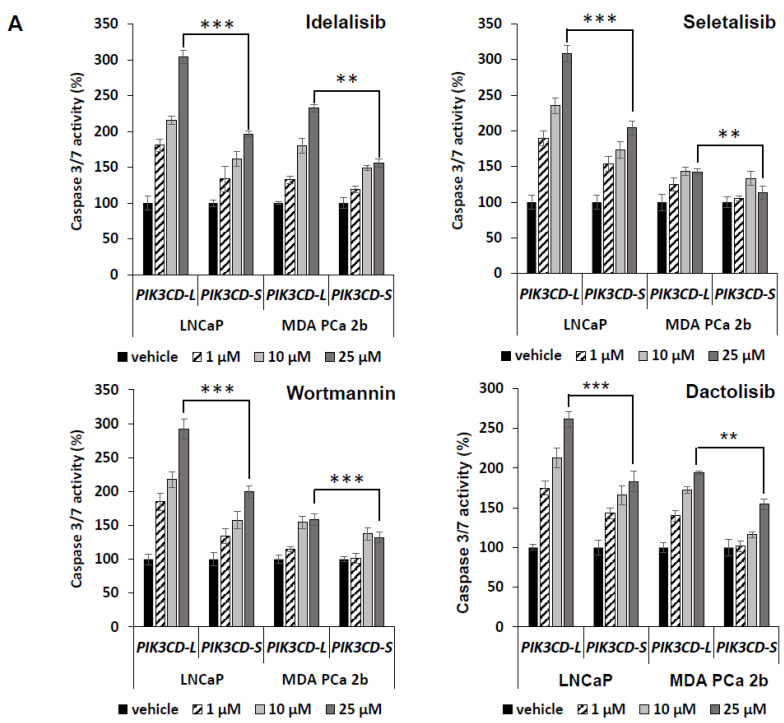

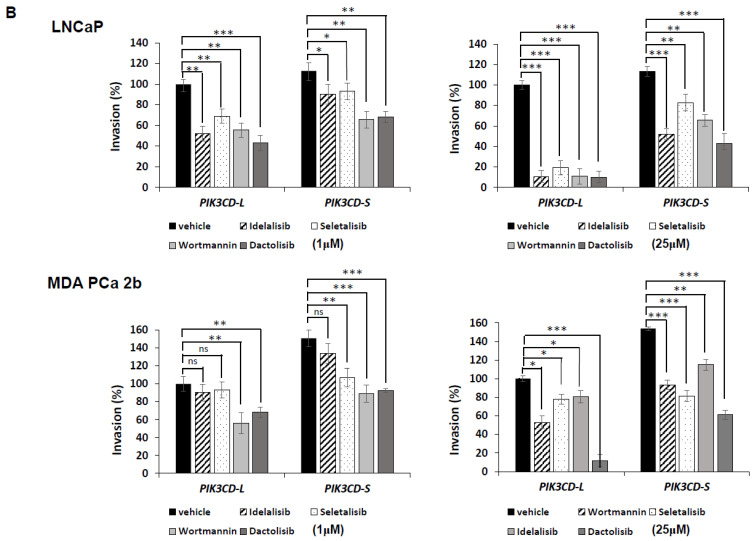
The full-length PI3Kδ-L is highly sensitive to the PI3K inhibitors, but PI3Kδ-S isoform exhibits resistance to the PI3K inhibitors. (**A**) Apoptosis activity assays, based on caspase 3/7 activities, in *PIK3CD-L*- and *PIK3CD-S*-expressing LNCaP and MDA PCa 2b cells under treatment of Idelalisib, Seletalisib, Wortmannin, and Dactolisib at 0, 1, 10, 25 µM. (**B**) Invasion assays in *PIK3CD-L*- and *PIK3CD-S*-expressing LNCaP and MDA PCa 2b cells under treatment of Idelalisib, Seletalisib, Wortmannin, and Dactolisib at 0, 1, 10, 25 µM. The data for both apoptosis and invasion assays were calculated from 3–4 independent experiments with duplicates, and the data were plotted as mean ± SEM. *^,^ **^,^ *** *p*-values (<0.05, 0.01, <0.001) were determined based on one-way ANOVA with Tukey’s post hoc test. ns: not significant.

Next, we tested whether these four drugs induce differential cell apoptotic manners in the *PIK3CD-L*- vs. *PIK3CD-S*-expressing PCa cells. As shown in Figure 3A, the *PIK3CD-L*-expressing LNCaP and MDA PCa 2b cells have exhibited an overall significantly higher cell apoptosis activity in response to the inhibitors, when compared to the *PIK3CD-S*-expressing LNCaP (*p*-value < 0.001) and MDA PCa 2b cells (*p*-value < 0.01 or <0.001) (Figure 3A). Moreover, *PIK3CD-L*-expressing LNCaP and MDA PCa 2b exhibited high sensitivities to the inhibitors in a dose-dependent manner. Specifically, 2.5- to 3-fold higher cell apoptotic activities were observed in *PIK3CD-L*-expressing LNCaP treated with 25 µM drug vs. vehicle. There were 1.4- to 2-fold increased apoptotic activities detected in *PIK3CD-L*-expressing MDA PCa 2b under drugs at 25 µM vs. vehicle, whereas ≤2-fold and ≤1.5-fold increases in cell apoptosis were detected in *PIK3CD-S*-expressing LNCaP and MDA PCa 2b treated with 25 µM drug vs. vehicle, respectively (Figure 3A). These results suggest that *PIK3CD-S*-expressing PCa is more antiapoptotic, compared to *PIK3CD-L*-expressing PCa, in response to the drugs.

Next, we performed cell invasion assays to assess how *PI3KCD-L* and *PI3KCD-S* affect the cell invasiveness in PCa. First, the *PIK3CD-L*- and *PIK3CD-S*-expressing LNCaP and MDA PCa 2b were treated with the same four drugs, followed by matrigel invasion assays. As anticipated, the four drugs exerted intermediate (35–55%) and potent (>80%) inhibitory effects on the *PIK3CD-L*-expressing LNCaP cells at 1 µM and 25 µM, respectively. In contrast, the four drugs exerted slight/intermediate (10–35%) and intermediate (20–45%) inhibitory effects on the *PIK3CD-S*-expressing LNCaP cells at 1 µM and 25 µM, respectively (Figure 3B, top panels). On the other hand, the *PIK3CD-L*-expressing MDA PCa 2b cells did not respond to Idelelisib nor Seletalisib at 1 µM and the invasive capacities were moderately inhibited (35–45%) by Wortmannin and Dactolisib at 1 µM (Figure 3B, top left panel). However, the cell invasion capacities of the *PIK3CD-L*-expressing MDA PCa 2b cells were moderately to significantly inhibited (20–90%) by all the drugs at 25 µM (Figure 3B, bottom right panel). Notably, the four drugs have demonstrated slight/intermediate (10–35%) and intermediate (25–55%) inhibitory effects on the cell invasion in the *PIK3CD-S*-expressing MDA PCa 2b cells at 1 µM and 25 µM, respectively (Figure 3B, bottom panel). In summary, we observed: (1) the *PIK3CD-S*-expressing cells have 15–20% higher invasive capacities than the *PIK3CD-L*-expressing cells in both the LNCaP and MDA PCa 2b backgrounds; (2) the MDA PCa 2b cells (endogenously expressing a higher *PIK3CD-S* level) have shown a generally higher resistance to the drug-mediated invasiveness inhibition, compared to the LNCaP cells (by comparing Figure 3B, bottom vs. top panels); and (3) the *PIK3CD-S*-expressing cells exhibit a higher resistance to the drug-mediated invasiveness inhibition, when compared to the *PIK3CD-L*-expressing cells (in both the LNCaP and MDA PCa 2b backgrounds). Taken all together, the cell apoptosis and invasion assay results have strongly suggested that the overexpression of the *PIK3CD-S* splice variant results in more oncogenic activities with enhanced antiapoptotic and invasive capacities.

### 3.5. Overexpression of PI3Kδ-S Splice Isoform Causes Resistance to Drug Inhibitory Effect on PI3K/AKT/Mtor Signaling

**Figure 4:**
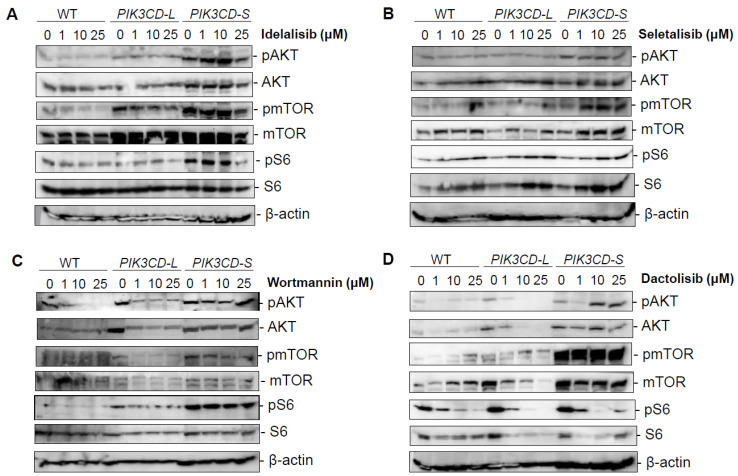
Inhibitory effects on PI3K/AKT/mTOR signaling in PCa cells overexpressing *PIK3CD-L* and *PIK3CD-S* variants in response to pan-PI3K, PI3Kδ, and PI3K/mTOR dual inhibitors. Western blot analysis of pAKT, AKT, pmTOR, mTOR, pS6, and S6 in MDA PCa 2b cells stably expressing *PIK3CD-L* and *PIK3CD-S* in presence of (**A**) Idelalisib, (**B**) Seletalisib, (**C**) Wortmannin, and (**D**) Dactolisib at concentrations of 0, 1, 10, and 25 μM. β-actin was served as an endogenous protein control.

To further investigate the molecular mechanisms underlying the drug resistance of PI3Kδ-S, a Western blot analysis was performed to examine the drug inhibitory effects on the PI3K-mediated signaling molecules in the PCa cells with differential PI3Kδ-L and PI3Kδ-S levels. The wild-type (WT) MDA PCa 2b and the MDA PCa 2b cells stably expressing *PIK3CD-L* and *PIK3CD-S* were grown and treated with four drugs (Idelalisib, Seletalisib, Wortmannin, and Dactolisib) at 0, 1, 10, and 25 µM. After the drug treatments for 48 h, the cells were harvested for the cell lysate preparation and then subjected to a Western blot analysis. First, the PI3Kδ-L and PI3K-S expression levels in the WT, *PIK3CD-L-,* and *PIK3CD-S*-expressing cells are shown in Supplementary Figure S1. The Western blot results confirmed that PI3Kδ-L and PI3Kδ-S were comparably expressed in the *PIK3CD-L*- and *PIK3CD-S*-expressing cells, while significantly higher than the WT cells. Second, a generalized reduction in the pAKT, pmTOR, and pS6 (as well as the total AKT, mTOR, and S6) was observed in the *PIK3CD-L*-expressing cells (but not in the *PIK3CD-S*-expressing cells) under the Idelalisib and Seletalisib treatments (Figure 4A,B). In the presence of Wortmannin (a pan-PI3K inhibitor), the *PIK3CD-L*-expressing cells were significantly inhibited in a dose-dependent manner, evident from the significantly decreased phosphorylation states of the AKT, mTOR, and S6 at 0, 1, 10, and 25 µM. In contrast, no inhibitory effects were observed in the *PIK3CD-S*-expressing cells, with no changes at the states of the pAKT, pmTOR, and pS6 at all concentrations (Figure 4C). As anticipated, Dactolisib (a dual PI3K/mTOR inhibitor) demonstrated a potent inhibitory effect on suppressing the pAKT, pmTOR, and pS6 (as well as the total AKT, mTOR, and S6) in the *PIK3CD-L*-expressing cells in a dose-dependent manner. In contrast, the *PIK3CD-S*-expressing cells have shown an excellent resistance to the inhibitory effect of Dactolisib on the pAKT and pmTOR at all concentrations. Intriguingly, Dactolisib demonstrated a dose-dependent inhibitory effect on pS6 and the total S6 in the *PIK3CD-S*-expressing cells (Figure 4D). These results perfectly reflect the pharmacological property of Dactolisib as a PI3K/mTOR dual inhibitor, for Dactolisib fails to inhibit the PI3Kδ-S-mediated phosphorylation of AKT but still effectively targets the mTOR and suppresses the mTOR-mediated phosphorylation of S6. Taken together, our Western blot results have confirmed that the PI3Kδ-S isoform is a more oncogenic form that is resistant (compared to PI3Kδ-L) with constitutively active PI3K/AKT signaling even in the presence of PI3K inhibitors.

### 3.6. Molecular Docking Provides a Molecular Insight into Binding Affinities of PI3Kδ-L and PI3Kδ-S with Pan-PI3K and PI3Kδ-Specific Inhibitors

Previous studies have shown that PI3Kδ is highly sensitive in responding to Idelalisib, Seletalisib, Wortmannin, and Dactolisib [25,27,28,29]. The PI3Kδ-specific inhibitor Idelalisib targets PI3Kδ with the highest affinity to the three binding sites at Glu826 (E826), Val828 (V828), and Ile910 (I910), located in the ATP-binding pocket of the PI3Kδ catalytic domain [31,32]. The energy of these interactions was achieved from favorable intermolecular interactions, including hydrogen bonding, hydrophobic interaction, van der Waals interaction, and ion pairing [33].

**Figure 5:**
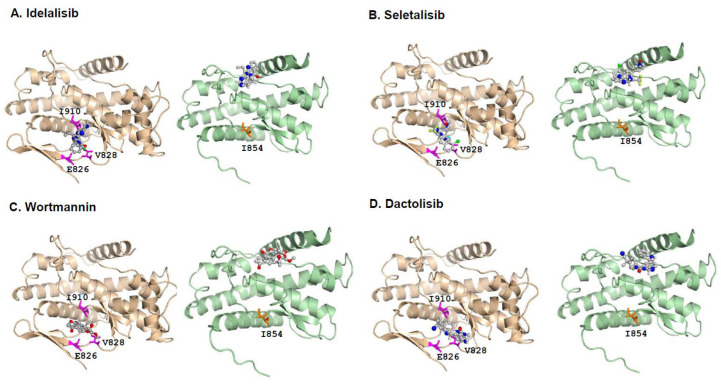

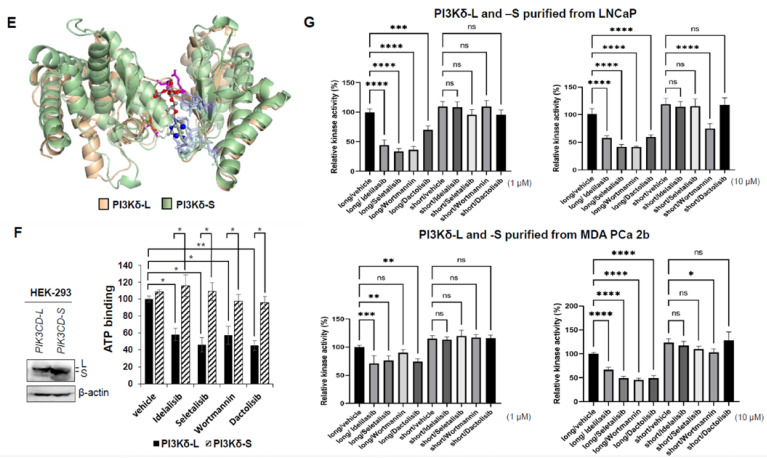
Molecular modeling of interactions between small-molecule inhibitors and PI3Kδ-L or PI3Kδ-S splice isoform. Molecular docking modeling using AutoDock Vina demonstrates the predicted physical interactions between PI3Kδ-L and PI3Kδ-S with (**A**) Idelalisib, (**B**) Seletalisib, (**C**) Wortmannin, and (**D**) Dactolisib. (**E**) Molecular modeling for ATP (ball-and-stick chemical) binding with PI3Kδ-L (brown) and PI3Kδ-S (green). (**F**) ATP-binding assays with PI3Kδ-L and PI3Kδ-S in competition with the four PI3K inhibitors (right panel). Western blot analysis (left panel) confirmed the comparable amounts of PI3Kδ-L and PI3Kδ-S were purified from *PIK3CD-L*- and *PIK3CD-S*-expressing HEK-293 cells, for the ATP-competitive assays. Data values were represented as mean ± SD from 3–4 independent experiments. *^,^ ** *p*-values (<0.05 and <0.01) were determined based on one-way ANOVA with Tukey’s post hoc test. (**G**) Cell-free PI3 kinase assays of purified PI3Kδ-L and PI3Kδ-S complexes incubated with four small-molecule inhibitors at 1 and 10 µM. The purified PI3Kδ-L and PI3Kδ-S complexes were from LNCaP and MDA PCa 2b cells transfected with pcDNA3.1-*PIK3CD-L*-V5-His and pcDNA3.1-*PIK3CD-S*-V5-His plasmids. **^,^ ***^,^ **** *p*-values (<0.01, <0.001, and <0.0001) were determined based on one-way ANOVA with Tukey’s post hoc test. ns: not significant. Data values represent mean ± SEM from 3–4 independent experiments with duplicated repeats.

To further investigate the binding affinities of small-molecule inhibitors with the PI3Kδ-L or PI3Kδ-S, molecular docking modeling was performed using Autodock 4.2.6 [34]. First, the three-dimensional (3D) structures of the PI3Kδ-L and PI3Kδ-S splice isoform were generated using SWISS-MODEL [35]. Second, the molecular docking modeling was conducted, and the results have confirmed a common region containing a core set of three amino acid residues (E826, V828, and I910) within the ATP-binding pocket of the PI3Kδ catalytic domain is crucial for efficient binding with the four inhibitors. All the inhibitors presented here were in physical contact with these three amino acid residues in the PI3Kδ-L catalytic domain (Figure 5A–D, left panels). Specifically, the docking modeling showed that Idelalisib interacts with PI3Kδ-L and formed hydrogen bonds with the V828 and I910 residues at distances of 3.6 Å and 3.3 Å, respectively, with a binding energy of −6.7 kcal/mol. Seletalisib formed hydrogen bonds with V828 and I910 at distances of 3.9 Å and 3.4 Å, respectively, with a binding energy of –7.4 kcal/mol. Wortmannin formed hydrogen bonds with E826, V828, and I910 at distances of 4.1 Å, 3.8 Å, and 3.4 Å, respectively, with a binding energy of –6.3 kcal/mol. Dactolisib formed a hydrogen bond with E826 and V828 at distances of 3.1 Å and 3.3 Å, respectively, with a binding energy of −7.3 kcal/mol. The molecular docking results were consistent with the previous crystallization and docking studies of the inhibitor bindings with the PI3K isoforms (α, γ, or δ) [31,32,36,37,38]. In contrast to the inhibitor docking to the PI3Kδ-L, the molecular docking results have shown that the four inhibitors have no physical contact with the amino acid residues located within the ATP-binding pocket of the PI3Kδ-S catalytic domain. Instead, the molecular docking modeling predicted that the inhibitors preferably target amino acid residues outside of the ATP-binding pocket of PI3Kδ-S, as indicated in Figure 5A–D (right panels). These off-target effects observed in the drug/PI3Kδ-S interactions are likely attributed to the missing hinge and the majority of the hydrophobic region in the catalytic domain of PI3Kδ-S (where Glu and Val residues are missing and only Ile is preserved, due to the skipping of exon 20). Taken together, the molecular docking results have strongly implicated that the differential affinities of Idelalisib, Seletalisib, Wortmannin, and Dactolisib with PI3Kδ-L or PI3Kδ-S may be due to the structural differences (with or without hinge/hydrophobic region) between these two kinase isoforms. Given the fact that these four drugs were designed as ATP-competitive inhibitors to PI3Kδ, the low affinities of the drugs with the ATP-binding pocket of PI3Kδ-S (based on the docking modeling) explains why the PI3Kδ-S splice isoform is more resistant to Idelalisib, Seletalisib, Wortmannin, and Dactolisib.

### 3.7. Competitive Drug/ATP-Binding Assays and Kinase Activity Assays Confirm a Reduced Binding Affinity between PI3Kδ-S and Drugs

The PI3 kinase activity is determined by the efficiency of ATP binding to PI3K. A previous study has revealed that ATP docks at K708, M752, D753, S754, W760, I777, and D911 in the ATP-binding pocket of the PI3Kδ catalytic domain [31]. In the presence of an ATP-competitive inhibitor (Idelalisib, Seletalisib, Wortmannin, or Dactolisib), ATP fails to efficiently dock at the binding sites in the catalytic domain, thereby hindering the PI3Kδ activity. We hypothesized that the differential drug sensitivities in PI3Kδ-L vs. PI3Kδ-S is mainly due to their differential ATP-binding affinities in the presence of the PI3K inhibitors. The molecular modeling by superimposing PI3Kδ-S with PI3Kδ-L and ATP docking demonstrated that (1) the 3D structures of PI3Kδ-L and PI3Kδ-S are nearly identical, except the hinge and hydrophobic regions are missing in PI3Kδ-S (Figure 5E and Figure S2), and (2) the ATP-binding sites are reserved in both the PI3Kδ-L and PI3Kδ-S isoforms; however, the critical drug-binding residues E826 and V828 are missing in the PI3Kδ-S catalytic domain (Supplementary Figure S2). For further validating our hypothesis and the molecular models, the ATP and drug were co-incubated with the PI3Kδ isoform (L or S) for determining the levels of the resulting PI3Kδ/ATP and PI3Kδ/drug complexes. In order to determine whether the PI3Kδ-L/ATP or PI3Kδ-S/ATP complex can be formed under competition with the PI3Kδ inhibitor in vitro, a competitive drug/ATP-binding assay was performed. First, the HEK-293 cells were transfected with *PIK3CD-L*- and *PIK3CD-S*-expressing plasmids, and the His-tagged PI3Kδ-L and His-tagged PI3Kδ-S proteins were purified by using Ni-NTA spin columns (Figure 5F, left panel and Supplementary Figure S3). Next, the purified His-tagged PI3Kδ-L (10 mM) or PI3Kδ-S (10 mM) was bound with Ni-NTA beads, and then inhibitors (10 mM) and ATP (1 mM) were added for incubation with Ni-NTA-bound PI3Kδ-L or PI3Kδ-S for 30 min at room temperature. After incubation, the bound Ni-NTA beads (with the His-tagged PI3Kδ/ATP or PI3Kδ/drug complex) were spun down, washed, and eluted for analysis. By examining the ATP concentrations in the eluted samples, a significantly lower ATP concentration was detected in the eluted His-tagged PI3Kδ-L complexes (PI3Kδ-L/ATP + PI3Kδ-L/drug) than the His-tagged PI3Kδ-S complexes (PI3Kδ-S/ATP + PI3Kδ-S/drug) (Figure 5F). Specifically, the ATP-binding affinity with PI3Kδ-L was drastically reduced in competition with Idelalisib, Seletalisib, Wortmannin, or Dactolisib (with 42%, 55%, 48%, and 58% decreases, respectively), compared to the vehicle control. In contrast, the ATP-binding affinities with PI3Kδ-S remained comparable in the presence of the drugs vs. vehicle (Figure 5F). In summary, the ATP-binding affinity with PI3Kδ-S is preserved while the ATP-binding affinity with PI3Kδ-L is greatly hampered, in the presence of the pan-PI3K or PI3Kδ inhibitors (ATP-competitive drugs). Importantly, the ATP-binding assay results have successfully validated, for the first time, the in silico molecular docking models presented in Figure 5A–D (drug binding with PI3Kδ-L and PI3Kδ-S) and Figure 5E (ATP binding with PI3Kδ-L and PI3Kδ-S).

Next, PI3 kinase activity assays were performed to assess the kinase activities of PI3Kδ-L and PI3Kδ-S under the drug treatments. First, the Ni-NTA-purified His-tagged PI3Kδ-L or PI3Kδ-S protein complexes from LNCaP and MDA PCa 2b (Supplementary Figure S3) were incubated with a small-molecule inhibitor (Idelalisib, Seletalisib, Wortmannin, or Dactolisib at 0, 1, and 10 µM), and then the reaction mixtures were subjected to the PI3 kinase activity assays. The kinase assay results have revealed that Idelalisib, Seletalisib, Wortmannin, and Dactolisib effectively suppressed the PI3Kδ-L activities (either purified from the LNCaP or MDA PCa 2b cells) at 1 µM and 10 µM concentrations (except the PI3Kδ-L from MDA PCa 2b at 1 µM Wortmannin). In contrast, no significant reduction in the kinase activities in PI3Kδ-S were observed under the drug treatments at 1 µM, and a slight-to-moderate reduction was observed in the kinase activity in PI3Kδ-S (purified either from the LNCaP or MDA PCa 2b cells) under the Wortmannin treatment at 10 µM (Figure 5G). Notably, the PI3Kδ-S complex has a higher basal kinase activity than the PI3Kδ-L complex, no matter if the complexes were purified from the *PIK3CD-S*-expressing LNCaP, MDA PCa 2b, or PC-3 cells (Figure 5G and Figure S4). These kinase assay results, consistent with the ATP-binding assays, suggest that PI3Kδ-S still preserves its normal kinase activity, even in the presence of drugs. In addition, the poor affinity between the drugs and PI3Kδ-S also interprets the drug resistance property observed in the *PIK3CD-S*-overexpressing cells.

### 3.8. Splicing Factor SRSF2 Modulates the Exon Skipping of PIK3CD

**Figure 6:**
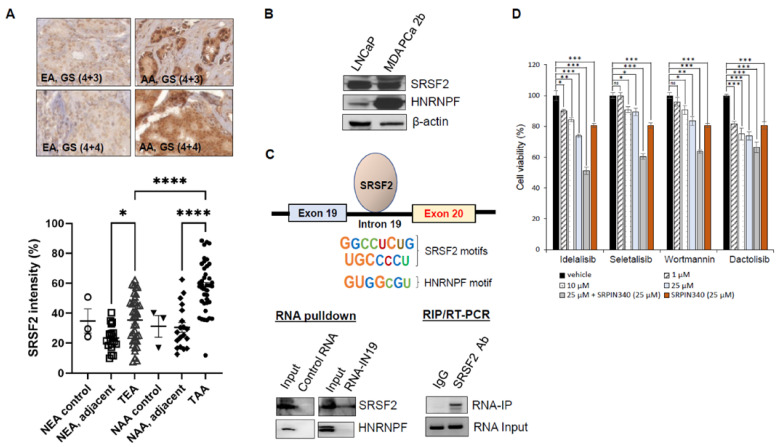
SRSF2 mediates skipping of exon 20 in *PIK3CD* pre-mRNA, and inhibition of SRPK1/2 sensitizes *PIK3CD-S*-expressing PCa to Idelalisib, Seletalisib, Wortmannin, and Dactolisib. (**A**) IHC staining of SRSF2 on a TMA containing 40–50 EA and AA PCa specimens. Representative IHC images of SRSF2 staining in EA and AA PCa with GS (4 + 3) and GS (4 + 4) are presented (top panel). Quantification of SRSF2 intensities in samples derived from NEA, NEA adjacent, TAA, NAA, NAA, NAA adjacent, and TAA. *p*-values (* <0.05, **** <0.0001) were determined based on one-way ANOVA with Tukey’s post hoc test. (**B**) Western blot analysis revealed that SRSF2 and HNRNPF were upregulated in MDA PCa 2b (AA PCa) vs. LNCaP (EA PCa) cells. (**C**) RNA pulldown and RIP/RT-PCR assays revealed that SRSF2 is the critical RNA-binding protein at the flanking intron adjacent to exon 20 of *PIK3CD* pre-mRNA. Two major SRSF2 and one HNRNPF putative binding motifs located in intron 19 were highlighted (top panel). Representative Western blot and gel images from RNA pulldown and RIP/RT-PCR assays were presented to confirm the SRSF2 binding at RNA fragment of flanking intron (RNA-IN19) covering the 2 SRSF2 and 1 HNRNPF motifs. (**D**) MTT assays for *PIK3CD-S*-expressing MDA PCa 2b in the presence of vehicle, small-molecule inhibitors (at 1, 10, and 25 µM), SRPIN340 (25 µM), and combination therapy (25 µM small-molecule inhibitor and 25 µM SRPIN340). *p*-values (* <0.05, ** <0.01, *** <0.001) were determined based on one-way ANOVA with Tukey’s post hoc test. ns: not significant. Data values represent mean ± SD from 4–5 independent experiments.

The exon profiling data revealed >2500 differential mRNA splicing events in AA PCa vs. EA PCa [14]. In the current study, we revisited the exon array data at the mRNA level, and six splicing genes (including *SRSF2*, *SRSF7*, *HNRNPF*, *HRNRPR*, *ISY1,* and *SF3B14*) were found upregulated in AA PCa vs. EA PCa (Supplementary Table S5). The RT-qPCR validation further confirmed that *SRSF2*, *SRSF7*, *HNRNPF*, and *HRNRPR* (but not *ISY1,* and *SF3B14*) were upregulated in the AA vs. EA PCa specimens (Supplementary Figure S5). We hypothesized that the overexpression of splicing factors may functionally promote the skipping of exon 20 in the *PIK3CD* pre-mRNA, resulting in the aberrant/oncogenic *PIK3CD-S* splice variant. The initial IHC assays have confirmed that the SRSF2, SRSF7, HNRNPF, and HNRNPR levels were all upregulated in AA PCa vs. EA PCa (Figure 6A and Figure S6). Furthermore, the quantification of the IHC assays on a TMA containing 42 EA PCa and 42 AA PCa have confirmed that SRSF2 is expressed significantly higher in AA PCa vs. EA PCa (Figure 6A, bottom panel). Although both the IHC and Western blot assays confirmed that SRSF2, SRSF7, HNRNPF, and HNRNPR were upregulated in AA PCa vs. EA PCa (Figure 6B and Figure S6), only the SRSF2- and HNRNPF-binding motifs were identified within intron 19 (a flanking intron upstream of *PIK3CD*) by using the SFmap program [39]. However, no binding motifs for SRSF7 and HRNRPR were identified in intron 19 (Supplementary Table S5). Given the results from the array data, RT-PCR validation, IHC staining, and Western blots and binding motif analyses, we hypothesized that SRSF2 and/or HNRNPF may serve as the critical splicing factor(s) targeting the flanking intron and then promoting the exon 20 skipping event, which results in the synthesis of *PIK3CD-S.* To validate this hypothesis, we conducted RNA pulldown and RIP/RT-PCR assays. First, an RNA fragment containing two SRSF2 and one HNRNPF-binding motifs in intron 19 (with the highest scores above the statistical cuffs, Supplementary Table S6, and Figure 6C, top panel) were synthesized. Second, the RNA-pulldown and RIP/RT-PCR assays were independently performed to validate whether SRSF2 or HNRNPF can be physically bound with the synthesized RNA fragment. The RNA pulldown assays have confirmed that SRSF2, but not HNRNPF, occupies the binding motifs of the synthesized RNA of the intron 19 fragment (Figure 6C, bottom left panel). Next, the RIP/RT-PCR assays were performed to further validate/confirm the RNA pulldown results. The RNA/nuclear protein complexes were immunoprecipitated with SRSF2 antibody and then followed by protein digestion, RNA purification, and RT-PCR reactions. The RIP/RT-PCR results have again demonstrated that the synthesized RNA fragment was bound by SRSF2 (Figure 6C, bottom right panel). Taken together, these data strongly suggest that SRSF2 physically binds to intron 19 and may play a functional role mediating the skipping of exon 20 in the *PIK3CD* pre-mRNA.

### 3.9. Inhibition of Splicing Kinase Sensitizes Pan-PI3Kδ and PI3Kδ-Selective Inhibitors against the PIK3CD-S-Expressing PCa

To develop a novel therapeutic strategy for overcoming the drug resistance conferred by the *PIK3CD-S* overexpression in AA PCa, we further explore the possibility of using a splicing inhibitor as a single agent or in combination with a PI3K/PI3Kδ inhibitor as synergistic drug therapy. Specifically, the *PIK3CD-S*-expressing MDA PCa 2b cells were treated with or without the PI3K/PI3Kδ inhibitor in the absence or presence of SRPIN340 (an SRPK1/2 inhibitor that blocks the phosphorylation of SRSF2) for 48 h, followed by an MTT assay to evaluate the cell viabilities. The MTT assay results have shown that the drug inhibits (from neglected to 30% for the four drugs) the cell viability in a dose-dependent manner. SRPIN340, as a single agent, exerts a moderate inhibitory capacity (~20% decrease in cell viability). However, combining the PI3K/PI3Kδ inhibitor with SRPIN340 resulted in an additive inhibitory effect with a significantly enhanced cytotoxicity (40–50% decrease in cell viability) in the *PIK3CD-S*-expressing MDA PCa 2b cells (Figure 6D). Taken together, these MTT results have suggested that SRPIN340 suppresses the SRSF2-mediated exon 20 skipping, consequently sensitizing the PI3K/PI3Kδ inhibitors with an enhanced cytotoxicity in the *PIK3CD-S*-expressing PCa cells.

## 4. Discussion

PI3Kδ is preferentially expressed in blood cells and is primarily considered as a critical oncogenic protein in lymphoid and myeloid malignancies [40,41,42]. In the past decade, emerging evidence has further indicated that PI3Kδ is also expressed in solid tumors, including breast cancer, colorectal cancer, liver cancer, PCa, melanoma, Merkel cell carcinoma, glioblastoma, and neuroblastoma [23,24,43]. It was found that PI3Kδ expression levels negatively correlate with the PTEN activity in breast cancer and PCa, and inhibiting PI3Kδ restores the PTEN activity and inhibits the AKT signaling and subsequently suppresses cell proliferation [23,44,45]. In addition, the PI3Kδ expression levels are correlated with cancer aggressiveness. For example, the PI3Kδ level increases during breast cancer progression from grade I to III, and an elevated PI3Kδ level correlates with advanced stage/metastasis in colorectal cancers and poor overall survival in HCCs [46]. Targeting PI3Kδ, therefore, has quickly emerged/been suggested as a promising therapy, either in monotherapy or in combination with immunotherapy, for treating solid tumors [24,47].

Target therapy using a PI3Kδ inhibitor, such as Idelalisib, is revolutionizing the treatment of CLL and NHL, the B-cell malignancies overexpressing PI3Kδ. Despite the high efficacy of Idelalisib as precision medicine for CLL/NHL patients, a fraction of patients developed resistance to Idelalisib. Yet the molecular mechanisms underlying the drug resistance remain unclear. To date, two mechanisms underlying the Idelalisib resistance were recently proposed. First, Sceffold et al. have revealed a non-genetic mechanism underlying the acquired resistance in CLLS under Idelalisib treatment. The PI3K/AKT inhibition by Idelalisib unexpectedly induces GSK3/FOXO1 activation, leading to the upregulation of IGF1R, which in turn activates the MAPK signaling in the Idelalisib-treated tumor cells. The inhibition of IGF1R using Linsitinib overcomes the Idelalisib resistance and induces cytotoxicity [48]. A more recent study identified a genetic mechanism, which echoes the critical role of upregulated MAPK signaling, in promoting Idelalisisb resistance in CLLs. Among the CLL patients who did not respond to Idelalisib therapy, 60% of the nonresponders have been identified with activating *MAP2K1*, *BRAF,* and *KRAS* mutations based on the whole-exome sequencing data. The overexpression of the activating *MAP2K1* mutation results in the upregulation of the MAPK/ERK signaling and confers resistance to Idelalisib. Notably, the inhibition of the MAPK signaling by the MEK1/2 inhibitor CI-1040 or the ERK1/2 inhibitor SCH772984 restores the sensitivity to Idelalisib in MEC1 cells with the activating *MAP2K1* mutation [49].

Besides the genetic (by activating the *MAP2K1* mutation) and non-genetic (Idelalisib-induced upregulation of MAPK signaling) mechanisms, the aberrant splicing of *PIK3CD* likely functions as another genetic driver for developing Idelalisib resistance. In cancers, accumulating evidence has highlighted aberrant mRNA splicing as a critical mechanism driving drug resistance. For instance, the aberrant mRNA splicing of *BCR-ABL, BIM, IKZF1, BRCA1, TP53, BRAF V600E, CD19, ERG, ERα,* and *AR* pre-mRNAs leads to the resistance of imatinib, the tyrosine kinase inhibitor (TKI), imatinib, the PARP inhibitor, cisplatin, vemurafenib, CART-19, taxanes, tamoxifen, and enzalutamide, respectively [18,20]. Our current study has further revealed that the SRSF2-mediated aberrant splicing of *PIK3CD* may represent a novel genetic mechanism driving the resistance to Idelalisib. Aberrant mRNA splicing within the *PIK3CD* pre-mRNA results in a short splice variant, *PIK3CD-S*, in which the exon 20 is skipped. This *PIK3CD-S* splice variant encodes PI3Kδ-S, an in-frame 104 kDa protein isoform lacking the hinge and the majority of the hydrophobic region in its catalytic domain. Our in silico molecular docking modeling has shown that the PI3Kδ-selective and pan-PI3K inhibitors perfectly dock in the ATP-binding pocket of the PI3Kδ-L catalytic domain, and the predicted drug-binding modes are consistent with the previous crystallization and molecular docking studies [31,32]. In contrast, our in silico docking modeling has strongly suggested that the drastic reduction in the binding affinity of Idelalisib, Seletalisib, Wortmannin, or Dactolisib with PI3Kδ-S is likely due to an off-target binding effect, where the drugs fail to target the ATP-binding pocket of the PI3Kδ-S catalytic domain (Figure 5A–D). The differential drug/kinase-binding modes between the drugs with PI3Kδ-L and PI3Kδ-S have been successfully validated by ATP/drug-competitive binding assays and cell-free kinase activity assays (Figure 5F,G). To date, this is the first report to validate the direct physical interactions and binding affinities of the ATP or drugs with the PI3Kδ splice isoforms. A further investigation of the *PIK3CD-S* and *PIK3CD-L* expression profiles in CLL and NHL patients may facilitate our understanding of the mechanisms conferring Idelalisib resistance (other than the IGF1R- and *MAP2K1* mutation-mediated MAPK/ERK activation).

Previous studies have revealed that PI3Kδ-selective inhibitors (Idelalisib, IC87114, and PIK-39) interact with PI3Kδ through contacting a core set of six active site residues (W760, E826, V827, V828, M900, and I910) in the ATP-binding pocket. The co-crystallization of PI3Kδ and Idelalisib/IC87114/PIK-39 demonstrated that the inhibitor binding induces an opening of a hydrophobic pocket in the PI3Kδ catalytic domain with high affinity [31,50]. Notably, V828 and I910 are in contact with the inhibitors, and E826 forms hydrogen bonds to the inhibitors [31]. PI3Kδ-S, a splice isoform lacking the hinge and the majority of the hydrophobic region, loses four active residues (V827, M900, and the core residues V828 and E828), significantly impairing the binding affinity between the drugs and PI3Kδ-S. Besides the missing portion of the hinge/hydrophobic region in the catalytic domain, the PI3Kδ-S catalytic domain exhibits a 3D structure nearly identical to the PI3Kδ-L catalytic domain (Figure 5E and Figure S2). The exon 20 skipping, surprisingly, does not affect any of the amino acid residues (K708, M752, D753, S754, W760, I777, and D911) critical for ATP binding in the PI3Kδ-S. The unique structure of the PI3Kδ-S isoform (lacking drug-binding residues but preserving ATP-binding sites) interprets why the ATP still effectively binds to PI3Kδ-S, even in the presence of ATP-competitive inhibitors. Further high-throughput screening for small-molecule inhibitors and/or developing new compounds that structurally fit into the ATP-binding pocket of PI3Kδ-S and exert potent inhibitory effects on PI3Kδ-S may warrant a novel therapeutic strategy for the Idelalisib-resistant CLLs/NHLs and solid tumors expressing *PIK3CD-S*.

Targeting splicing machinery has been proposed as a promising approach in treating cancers with aberrant splice variants. SRPIN340 and SRPIN-1 have been shown to effectively target SRPK1/2, which leads to inhibiting the phosphorylation of SR proteins [18,20,51]. In human cells, SRSF2 has been implicated in promoting both in vivo exon skipping and inclusion events [52]. A recent RNA-seq study further revealed that SRSF2 binding in flanking constitutive exon often leads to exon skipping in human hepatocellular carcinoma (HCC) [53]. The upregulation of SRSF2 (Figure 6A) and the multiple SRSF2-binding motifs in the flanking intron (Supplementary Table S6) appear to fulfill the requirement for achieving the skipping of exon 20 from the *PIK3CD* pre-mRNA. Targeting SRSF2, theoretically, could reverse the aberrant splicing and suppress the synthesis of *PIK3CD-S*. Indeed, a combination of the inhibition of SRSF2 by SRPIN340 sensitizes the resistant PCa cells to PI3Kδ and PI3K inhibitors (Figure 6D). This novel synergistic therapy opens a new opportunity for effectively treating PI3Kδ-S-expressing or Idelalisib-resistant tumors. In summary, our study has provided a first molecular insight into the PI3Kδ-S-driven drug resistance to Idelalisib, Seletalisib, Wortmannin, and Daclisib. Moreover, this study highlights a novel SRSF2-mediated RNA splicing mechanism for the synthesis of the aberrant *PIK3CD-S* variant, which encodes a splice isoform conferring drug resistance.

## 5. Conclusions

To date, this is the first comprehensive study to validate the *PIK3CD-S/PIK3CD-L* profiles in a panel of PCa patients and cell lines, evaluate the functional impacts of *PIK3CD-L* and *PIK3CD-S* in PCa invasiveness/apoptosis, perform the molecular docking of drugs with PI3Kδ-L and PI3Kδ-S, validate the physical interaction of the ATP/drug with PI3Kδ-L and PI3Kδ-S, identify the SRSF2-mediated splicing mechanism for *PIK3CD-S* synthesis, and develop a novel synergistic drug therapy to overcome the Idelalisib resistance in AA PCa.

In conclusion, a deep understanding of splicing-driven drug resistance mechanisms, a novel synergistic strategy combining PI3K and SRPK1/2 inhibitors, and further screening/designing novel compounds targeting PI3Kδ-S may pave a new path for precision medicine for resistant AA PCa and other hematologic/non-hematologic tumors overexpressing *PIK3CD-S*.

## Data Availability

The data presented in this study are available upon request from the corresponding author.

## Supplementary Materials

The following supporting information can be downloaded at: https://www.mdpi.com/article/10.3390/cancers15041337/s1, Table S1: Primer sequences for RT-PCR reaction to amplify *PIK3CD-L*, *PIK3CD-S*, and endogenous control EIF1AX transcripts; Table S2: Amino acid sequences for the catalytic domains of PI3Kδ-L and PI3Kδ-S isoforms; Table S3: Primer sequences for RT-PCR reactions followed by the RNA-IP (RIP) assays; Table S4: IC_50_ values for the PCa cell lines under treatment of Idelalisib, Seletalisib, Wortmannin, and Dactolisib; Table S5: Differentially expressed splicing factors identified from microarray profiling data in AA PCa vs. EA PCa specimens; Table S6: Putative binding motifs of SRSF2 and HNRNPF located at intron 19 of *PIK3CD* pre-mRNA; Figure S1: Western blot analysis to examine the expression levels of His-tagged PI3Kδ-L and PI3Kδ-S in MDA PCa 2b cells; Figure S2: 3D structures of PI3Kδ-L and PI3Kδ-S catalytic domains; Figure S3: Purification of PI3Kδ-L and PI3Kδ-S from HEK293, LNCaP, and MDA PCa 2b cells; Figure S4: Cell-free kinase activity assays of PI3Kδ-L and PI3Kδ-S isoforms; Figure S5: RT-qPCR validation of SRSF2, SRSF7, HNRNPF, HNRNPR, ISY1 and SF3B14 expression levels; Figure S6: IHC and western blot assays to verify protein expression levels of SRSF2, SRSF7, HNRNPF, and HNRNPR in AA and EA PCa.
