# Supplementary material for: Molecular Insight into Drug Resistance Mechanism Conferred by Aberrant PIK3CD Splice Variant in African American Prostate Cancer
Source: Cancers (Basel). 2023 Feb 20;15(4):1337. doi: 10.3390/cancers15041337 (PMC9954641; doi:10.3390/cancers15041337)
Supplement: Supplementary file 1 [file cancers-15-01337-s001.zip › Supplementary Figures.pdf]

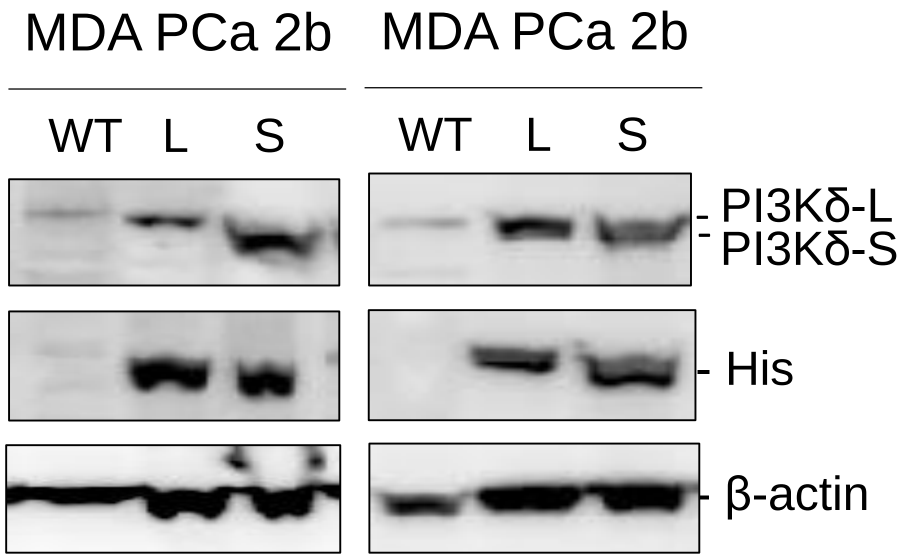

Supplementary Figure S1. Western blot analysis to examine the expression levels of His-tagged PI3Kδ-L and PI3Kδ-S in MDA PCa 2b cells. Two sets of representative MDA PCa 2b wild-type (WT), MDA PCA 2b stably expressing *PIK3CD-L* (L), and MDA PCA 2b stably expressing *PIK3CD-S* (S) cells were harvested, lysed using M-PER lysis buffer for preparation of protein lysates. The cell lysates were subjected to western blot analysis, and the His-tagged PI3Kδ-L and PI3Kδ-S levels were detected using monoclonal mouse antibody against PI3Kδ and monoclonal rabbit antibody against His tag. β-actin was used as an endogenous protein control.

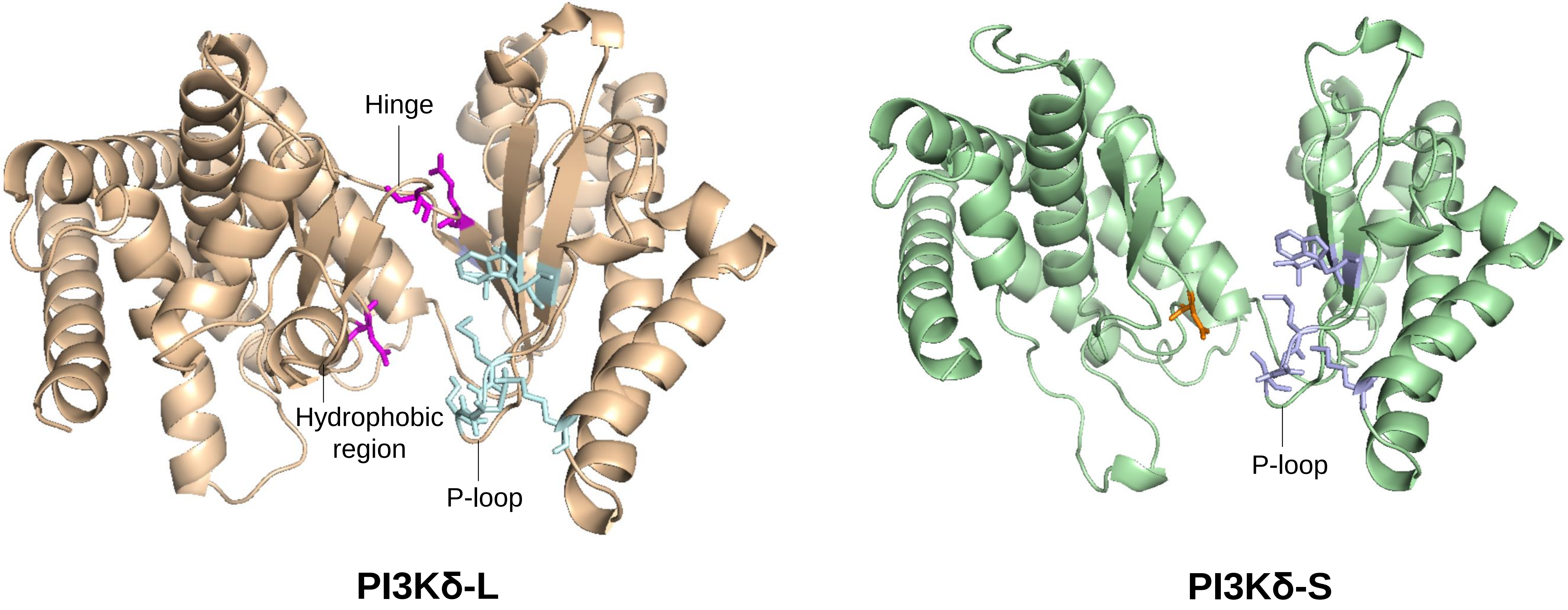

Supplementary Figure S2. 3D structures of PI3Kδ-L and PI3Kδ-S catalytic domains. Molecular modeling shows that PI3Kδ-L and PI3Kδ-S have nearly identical 3D conformations except that hinge and majority of hydrophobic region are missing in PI3Kδ-S. P-loop and the all ATP binding residues (K708, M752, D753, S754, W760, and I777, indicated as blue and purple in PI3Kδ-L and PI3Kδ-S) are preserved in both PI3Kδ-L and PI3Kδ-S. However, drug binding sites resided at hinge (E826 and V828, pink residues located at hinge of PI3Kδ-L) are missing in PI3Kδ-S, and only one residue Ile (orange residue) is preserved in PI3Kδ-S isoform.

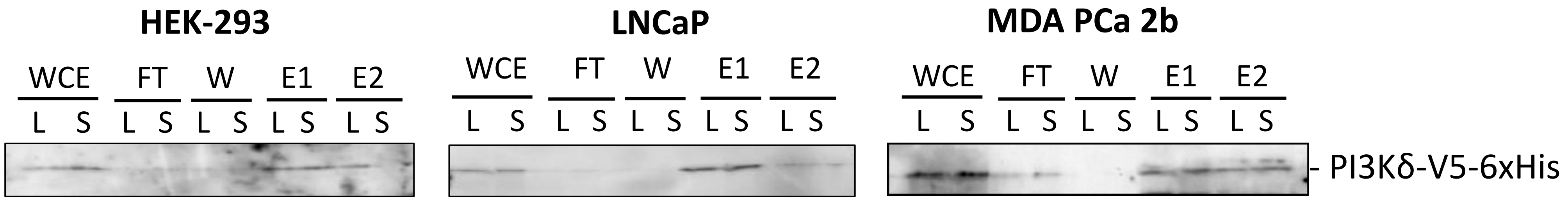

Supplementary Figure S3. Purification of PI3Kδ-L and PI3Kδ-S from HEK293, LNCaP, and MDA PCa 2b cells. The HEK293, LNCaP and MDA PCa 2b expressing *PIK3CD-L* and *PIK3CD-S* were harvested, lysed for preparation of cell lysates. The His-tagged PI3Kδ-L and PI3Kδ-S isoforms were purified from the collected cell lysates using Ni-NTA spin columns. The whole cell extract (WCE), flow-through (FT), wash (W), and eluted (E1 and E2) samples were subjected to western blot analysis. Monoclonal mouse PI3Kδ antibody was used to detect the His-tagged PI3Kδ-L and PI3Kδ-S isoforms.

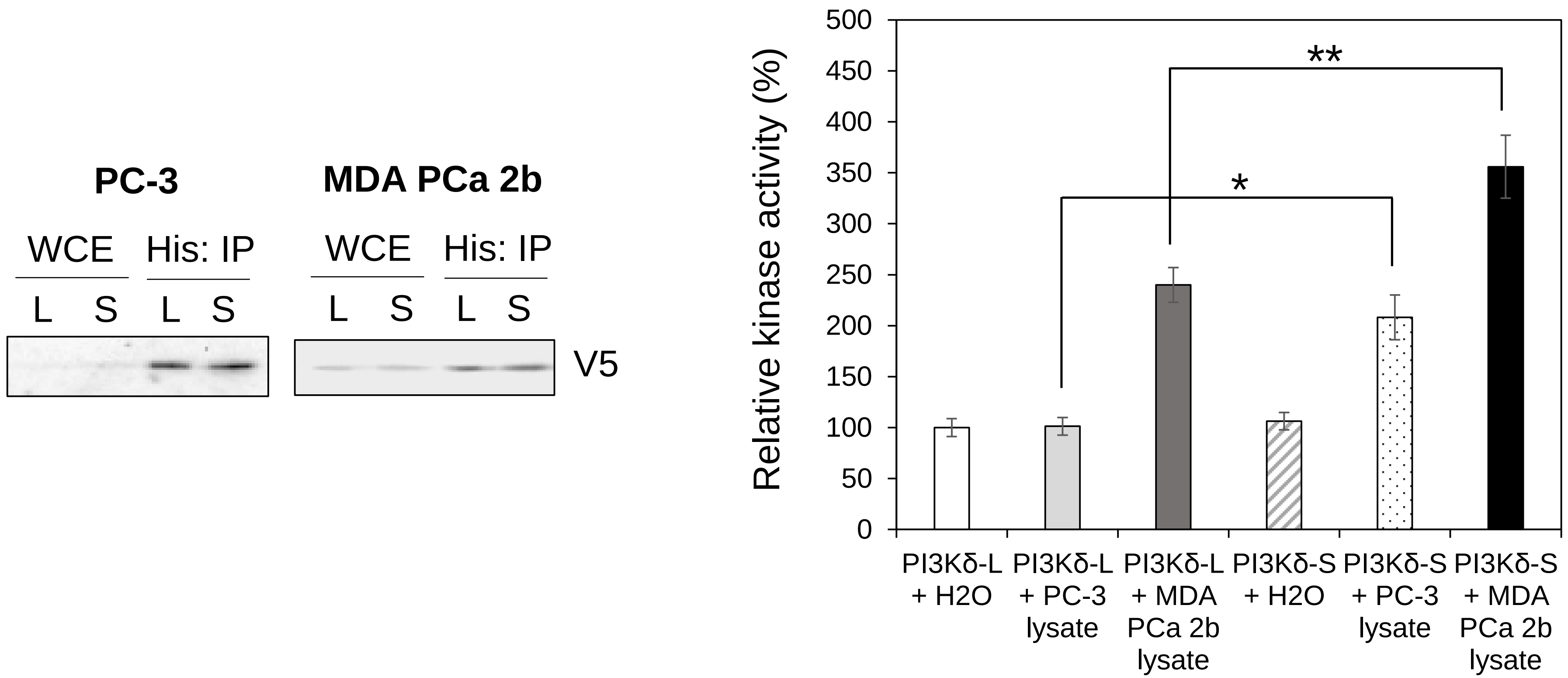

Supplementary Figure S4. Cell-free kinase activity assays of PI3Kδ-L and PI3Kδ-S isoforms. (A) PI3Kδ-L and PI3Kδ-S isoforms were purified by immunoprecipitation (IP) of *PIK3CD-L-V5-6×HIS* (L) and *PIK3CD-S-V5-6×HIS* (S) expressing PC-3 and MDA PCa 2b lysates using anti-His antibody and protein A/G beads. Western blot using anti-V5 antibody confirmed that PI3Kδ-L-V5-6×His and PI3Kδ-S-V5-6×His were purified. **(B)** PI3 kinase activities of purified PI3Kδ-L and PI3Kδ-S (mixed with H_2O, PC-3 cell lysate, or MDA PCa 2b cell lysate) were measured. Kinase activity of PI3Kδ-L mixed with H_2O was defined as 100% for normalization. *p<0.05 and **p<0.05, significant different kinase activities in PI3Kδ-S vs. PI3Kδ-L in presence of PC-3 lysates and MDA PCa 2b lysates, respectively. Data values represent mean ± SD, from 3-4 independent experiments.

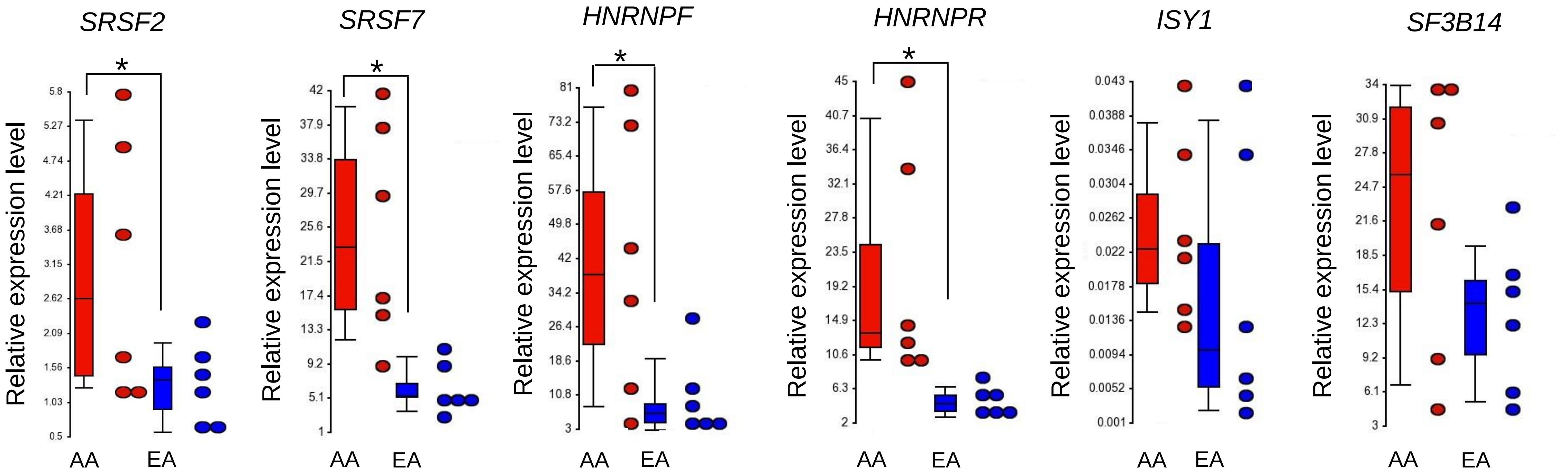

Supplementary Figure S5. RT-qPCR validation of *SRSF2, SRSF7, HNRNPF, HNRNPR, ISY1* and *SF3B14* expression levels. RNA samples purified from 6 AA and 6 EA PCa patient samples were used for RT-qPCR assays to examining the gene expression levels of the six splicing factors identified differentially expressed from microarray data. *p<0.05, significant different expression levels in AA PCa vs. EA PCa, based on two-tailed student t-test analysis.

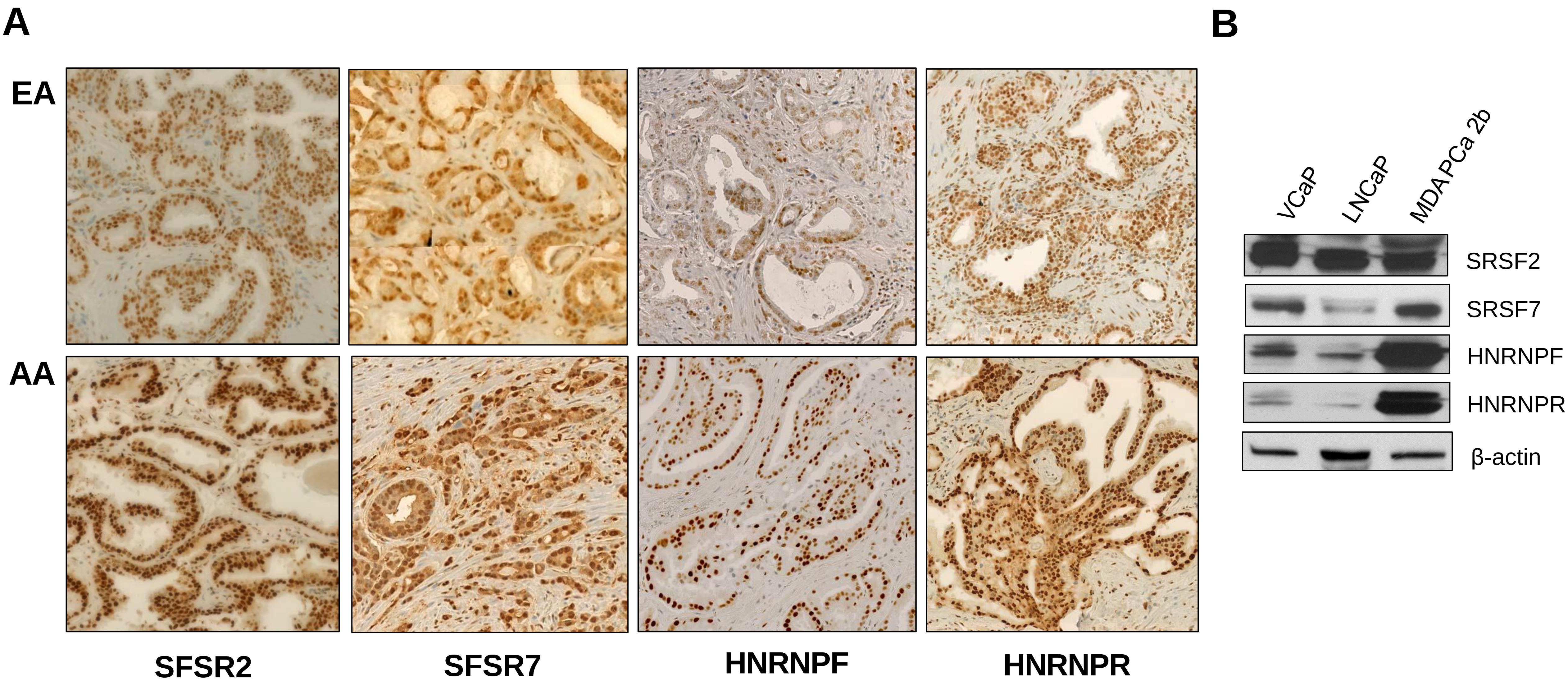

Supplementary Figure S6. IHC and western blot assays to verify protein expression levels of SRSF2, SRSF7, HNRNPF, and HNRNPR in AA and EA PCa. (A) Representative IHC staining images of the SRSF2, SRSF7, HNRNPF, and HNRNPR levels in AA PCa vs. EA PCa. **(B)** Western blot analysis of SRSF2, SRSF7, HNRNPF, and HNRNPR levels in EA PCa (VCaP and LNCaP) vs. AA PCa (MDA PCa 2b) cells. β-actin was served as endogenous protein control for western blot analysis.
