# Supplementary material for: Molecular Insight into Drug Resistance Mechanism Conferred by Aberrant PIK3CD Splice Variant in African American Prostate Cancer
Source: Cancers (Basel). 2023 Feb 20;15(4):1337. doi: 10.3390/cancers15041337 (PMC9954641; doi:10.3390/cancers15041337)
Supplement: Supplementary file 1 [file cancers-15-01337-s001.zip › Supplementary Tables S1-S6.pdf]

Supplementary Table S1. Primer sequences for RT-PCR reaction to amplify *PIK3CD-L*, *PIK3CD-S*, and endogenous control EIF1AX transcripts.

Primer ID	Sequences of primer (5' to 3')
PIK3CD-f	CTGAGCTCTCAGAAGACC
PIK3CD-r	GCTCGCGGTTGATTCCAA
EIF1AX-f	GTACTGGAGAGGGGAGAGCA
EIF1AX-r	TGAAGCTGAGACAAGCAGGA

Supplementary Table S2. Amino acid sequences for the catalytic domains of PI3Kδ-L and PI3Kδ-S isoforms.

**Sequences for PI3Kδ-L catalytic domain (268 amino acid residues)**
VGIIFKNGDDLRQDMLTLQMIQLMDVLWKQEGLDLRMTPYGCLPTGDRTGLIEVVLRSDTIANIQLNKSNM AATAAFNKDALLNWLKSKNPGEALDRAIEEFTLSCAGYCVATYVLGIGDRHSDNIMIRESGQLFHIDFGHF LGNFKTKFGINRERVPFILTYDFVHVIQQGKTNNSEKFERFRGYCERAYTILRRHGLLFLHLFALMRAAGLP ELSCSKDIQYLKDSLALGKTEEEALKHFRVKFNEALRESWKTKVNWLAHNVSKD

**Sequences for PI3Kδ-S catalytic domain (212 amino acid residues)**
VGIIFKNGDDLRQDMLTLQMIQLMDVLWKQEGLDLREALDRAIEEFTLSCAGYCVATYVLGIGDRHSDNIM IRESGQLFHIDFGHFLGNFKTKFGINRERVPFILTYDFVHVIQQGKTNNSEKFERFRGYCERAYTILRRHGL LFLHLFALMRAAGLPELSCSKDIQYLKDSLALGKTEEEALKHFRVKFNEALRESWKTKVNWLAHNVSKD

Supplementary Table S3. Primer sequences for RT-PCR reactions followed by the RNA-IP (RIP) assays. The primers were designed to verify whether SRSF2 or HNRNPF binds to the synthesized RNA fragment containing putative binding motifs.

**Primer ID**	**Sequences of primer (5' to 3')**
PIK3CD-IN19-f	GCCCATCTTGGGAAGCAGTGG
PIK3CD-IN19-r	CTTGGAAGAGAACCAGACAGA

Supplementary Table S4. IC_{50} values for the PCa cell lines under treatment of Idelalisib, Seletalisib, Wortmannin, and Dactolisib. The IC_{50} values were determined based on the dose-response curves presented in Figure 2A.

	IC_{50} (μM)			
**PCa cell line**	**Idelalisib**	**Seletalisib**	**Wortmannin**	**Dactolisib**
22Rv1	1.1	15.7	36.2	9.2
LNCaP	8.2	3.7	0.4	0.2
PC-3	80.2	85.5	46.5	0.2
RC77 T/E	17.2	176.7	121.3	16.4
MDA PCa 2b	132.2	53.1	1.4	35.9

Supplementary Table S5. Differentially expressed splicing factors identified from microarray profiling data in AA PCa vs. EA PCa specimens. RNA samples purified from 20 AA PCa and 15 EA PCa were subjected to exon array analysis, and the mRNA level data identified six splicing factors were upregulated in AA PCA vs. EA PCa. The transcript cluster IDs, gene symbols, RefSeq IDs, ratios, fold changes, and regulation directions were listed.

Transcript Cluster ID	Gene Symbol	RefSeq	Ratio (AA vs. EA PCa)	Fold-Change (AA vs. EA PCa)	Regulation	Enriched/ depleted mRNAs
3771800	SRSF2	NM_003016	1.74536	1.74536	AA PCa up vs EA PCa	AA-enriched
2548970	SRSF7	NM_001031684	1.87885	1.87885	AA PCa up vs EA PCa	AA-enriched
3286286	HNRNPF	NM_004966	1.89991	1.89991	AA PCa up vs EA PCa	AA-enriched
2401275	HNRNPR	NM_001102398	1.77853	1.77853	AA PCa up vs EA PCa	AA-enriched
2694617	ISY1	NM_020701	1.74069	1.74069	AA PCa up vs EA PCa	AA-enriched
2544179	SF3B14	NM_016047	1.72692	1.72692	AA PCa up vs EA PCa	AA-enriched

Supplementary Table S6. Putative binding motifs of SRSF2 and HNRNPF located at intron 19 of *PIK3CD* pre-mRNA. The sequence analysis and binding motif prediction were performed using SFmap program (http://sfmap.technion.ac.il/index.html). The two SRSF2 motifs (sequence positions at 285 and 269) and 1 HNRNP motif (sequence position at 268) that show top scores significantly higher than their cutoffs were selected for the RNA pulldown and RIP/RT-PCR assays in this study.

Splicing Factor: SRSF2 (SC35), Motif: gryymcyr, Cutoff: 0.600

Sequence Position	Genomic Coordinate	K-mer	Score
6	chr1:9782670	gaccccca	0.681
**285**	chr1:9782949	**ggccucug**	**0.706**

Splicing Factor: SRSF2 (SC35), Motif: ugcygyy, Cutoff: 0.586

Sequence Position	Genomic Coordinate	K-mer	Score
57	chr1:9782721	ugccccu	0.656
184	chr1:9782848	ugcuccu	0.602
214	chr1:9782878	uguugcu	0.662
234	chr1:9782898	ugccccu	0.695
**269**	chr1:9782933	**uggcguc**	**0.695**
389	chr1:9783053	ugcuuuu	0.626
485	chr1:9783149	ugccauu	0.687

Splicing Factor: HNRNPF, Motif: gukgykg, Cutoff: 0.586

Sequence Position	Genomic Coordinate	K-mer	Score
31	chr1:9782695	gugucug	0.653
73	chr1:9782737	cuuguug	0.597
76	chr1:9782740	guugaag	0.597
**268**	chr1:9782932	**guggcgu**	**0.672**

Splicing Factor: hnRNPF, Motif: gggug, Cutoff: 0.700

Sequence Position	Genomic Coordinate	K-mer	Score
81	chr1:9782745	aggug	0.737
